# Fibrin-Binding Peptide-Functionalized H_2_O_2_-Responsive Retinoic Acid Micelles for Attenuating Thrombosis-Associated Oxidative and Inflammatory Responses

**DOI:** 10.3390/biomedicines14092015

**Published:** 2026-09-08

**Authors:** Junkai Zhao, Mengting Xie, Jianghao Yu, Ran Yan, Yue Wang

**Affiliations:** Key Laboratory of Biomedical Functional Materials, School of Science, China Pharmaceutical University, No. 639 Longmian Avenue, Jiangning District, Nanjing 211198, China; 2020220547@stu.cpu.edu.cn (J.Z.);

**Keywords:** thrombotic disease, retinoic acid prodrug, polymer micelle, hydrogen peroxide, computer-aided drug design

## Abstract

**Background/Objectives**: Thrombotic cardiovascular diseases remain a major cause of morbidity and mortality worldwide. Current antithrombotic therapies are limited by insufficient thrombus targeting. This study aimed to develop a fibrin-binding peptide-functionalized, hydrogen peroxide (H_2_O_2_)-responsive polymeric micelle and to evaluate its physicochemical properties and biological effects under H_2_O_2_-induced endothelial oxidative stress and preliminary FeCl_3_-induced thrombosis conditions. **Methods**: A fibrin-binding peptide, P2 (VTFIKC), was screened using computer-aided drug design and evaluated through microscale thermophoresis and in vitro thrombus adhesion assays. An all-trans retinoic acid (atRA)-based boronate ester prodrug, BORA, was synthesized to enable H_2_O_2_-triggered degradation and drug release. BORA was co-assembled with P2-modified Mal-PEG-b-PAsp to prepare P2-Mal-PEG-b-PAsp/BORA micelles. Their physicochemical properties, H_2_O_2_ responsiveness, H_2_O_2_-scavenging activity, cytocompatibility, cytoprotective effects, anti-inflammatory activity, and preliminary in vivo efficacy were evaluated. **Results**: The resulting micelles exhibited a suitable nanoscale size, acceptable cytocompatibility, H_2_O_2_-responsive changes in particle size distribution, and concentration-dependent H_2_O_2_-scavenging activity. In H_2_O_2_-stimulated human umbilical vein endothelial cells, micelle treatment was associated with improved cell viability, lower intracellular ROS-associated fluorescence, and reduced TNF-α and IL-1β concentrations. In a FeCl_3_-induced rat carotid artery thrombosis model, P2-Mal-PEG-b-PAsp/BORA micelles altered platelet- and leukocyte-related hematological indices and exhibited preferential accumulation in the thrombotic carotid artery. **Conclusions**: P2-Mal-PEG-b-PAsp/BORA micelles combine P2-mediated fibrin-binding potential, H_2_O_2_-responsive release behavior, and H_2_O_2_-scavenging activity. The findings provide preliminary support for further investigation of this peptide-functionalized nanoplatform.

## 1. Introduction

Thrombotic cardiovascular diseases, including myocardial infarction, ischemic stroke, coronary artery disease, and pulmonary embolism, remain among the leading causes of morbidity and mortality worldwide [1]. The formation of pathological thrombi can obstruct blood flow, induce local ischemia and oxidative injury, and eventually lead to severe organ dysfunction [2,3]. During thrombogenesis, vascular endothelial injury exposes subendothelial matrix components and promotes platelet adhesion, activation, and aggregation. Activated platelets subsequently release multiple pro-thrombotic mediators, recruit additional platelets, and cooperate with fibrin networks to stabilize thrombotic structures [4].

Current pharmacological strategies for thrombotic diseases include anticoagulants, antiplatelet agents, and thrombolytic drugs [5], while their applications are restricted by poor thrombus specificity; moreover, the ability of conventional antithrombotic agents to modulate the pathological thrombus microenvironment is relatively poor [5,6,7]. These challenges highlight the need for targeted therapeutic systems capable of localizing at thrombotic sites and improving the local pathological microenvironment.

The thrombus microenvironment is characterized by abundant fibrin, activated platelets, and elevated levels of reactive oxygen species (ROS) [3,8,9]. H_2_O_2_ is a stable ROS in the vascular system, which plays an important role in platelet activation, platelet–endothelial interactions, and inflammatory responses. Excessive H_2_O_2_ can aggravate endothelial injury, amplify platelet activation, and promote the secretion of inflammatory cytokines, thereby contributing to the progression of thrombotic vascular diseases [10,11,12]. Recent megakaryocyte transcriptomic analysis further demonstrated age- and sex-specific changes in programs related to platelet activation, inflammation, mitochondrial function, and oxidative stress, underscoring the biological heterogeneity connecting redox regulation with thrombotic potential [13]. Therefore, scavenging H_2_O_2_ and attenuating oxidative stress may be an effective strategy for suppressing platelet activation and alleviating thrombosis-associated oxidative and inflammatory injury.

Nanomedicine-based drug delivery systems, including polymeric micelles, liposomes, and other nanoscale carriers, can improve drug solubility, prolong systemic circulation, and reduce off-target toxicity [14,15]. Moreover, the surface functionalization of nanocarriers with thrombus-targeting ligands enables selective accumulation at thrombotic sites [15]. Among various thrombus-associated targets, fibrin is particularly attractive owing to its abundance, structural importance within thrombi, and extensive distribution throughout the clot network [16,17]. Peptide ligands are well-suited for nanocarrier functionalization due to their small molecular size, low immunogenicity, ease of synthesis, and flexible chemical modifiability [18,19]. Nevertheless, the practical application of many thrombus-targeting peptides remains challenged by suboptimal proteolytic stability, binding affinity, or in vivo targeting performance [20,21]. In this study, hexapeptides, due to their practical balance of structural simplicity, synthetic feasibility, and sufficient side-chain diversity for fibrin-pocket recognition, were selected as an initial focus for CADD-guided screening [22,23]. All-trans retinoic acid (atRA), a biologically active metabolite of vitamin A, has been reported to exhibit anti-inflammatory, antioxidant, antiplatelet, fibrinolytic, and anti-atherosclerotic functions, indicating its potential value in the treatment of thrombotic diseases [24,25]. However, the clinical translation of atRA for cardiovascular applications is restricted by its poor aqueous solubility, limited stability, and unsatisfactory bioavailability [26]. Prodrug and nanocarrier strategies may mitigate these limitations by improving its physicochemical properties and enabling controlled release at pathological sites. Boronate ester linkages, which can be selectively oxidized by H_2_O_2_ in ROS-rich environments, are widely employed as H_2_O_2_-responsive chemical motifs [27,28]. Incorporating a boronate ester structure into an atRA-based prodrug may enable H_2_O_2_-triggered drug release while simultaneously consuming excessive H_2_O_2_. Furthermore, polymeric micelles were selected due to their amphiphilic core–shell structure, which is suitable for enhancing aqueous solubilization of poorly water-soluble atRA and its hydrophobic prodrug BORA [29,30]. More broadly, the recent nanotechnology literature has emphasized that surface engineering and stimulus-responsive designs can improve drug localization and controlled release, while also highlighting the need for rigorous evaluation of biodistribution, efficacy, and safety before clinical translation [31].

**Figure 1:**
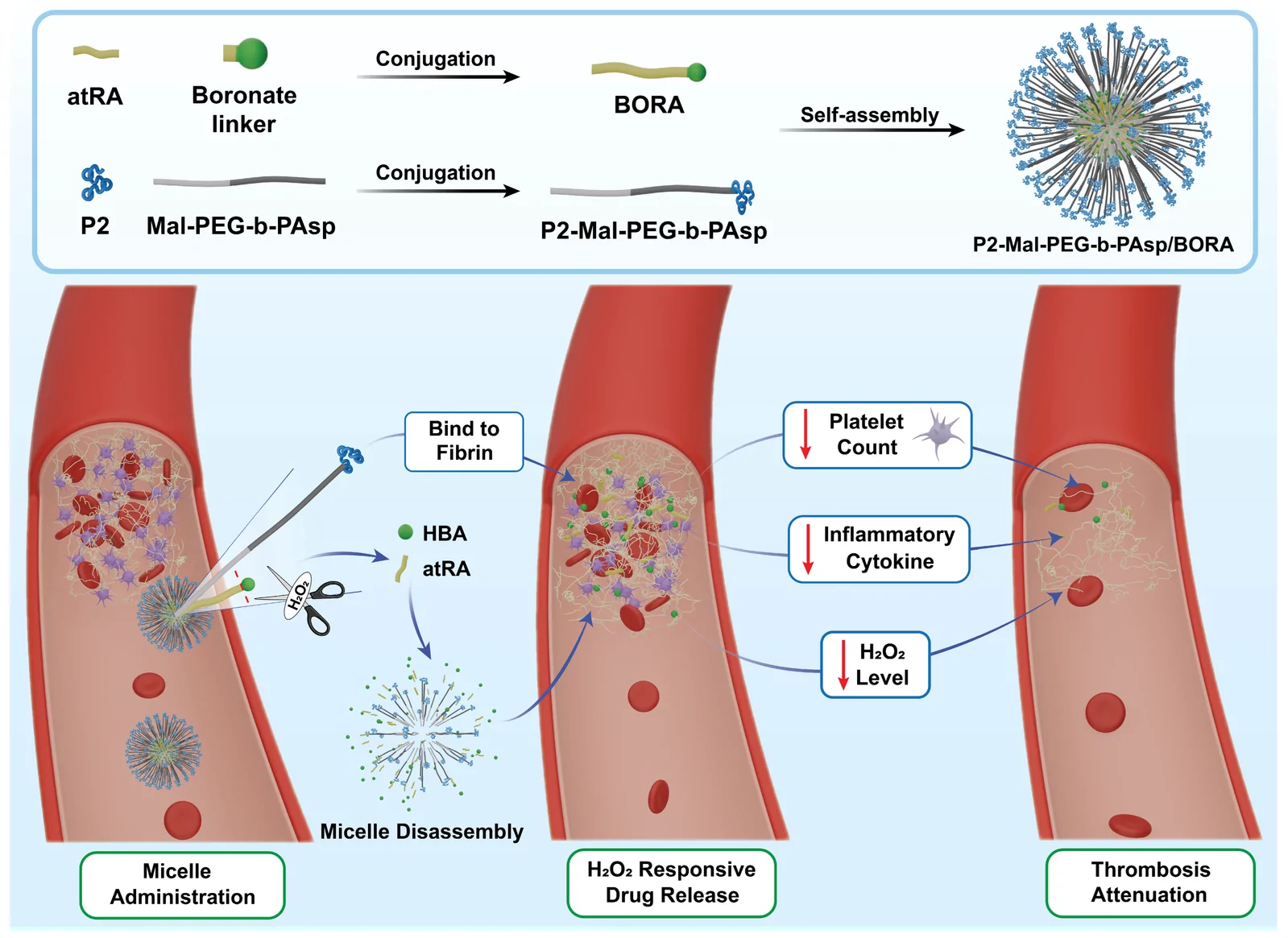
Schematic illustration of the preparation, H_2_O_2_-responsive drug-release behavior, and proposed biological effects of P2-Mal-PEG-b-PAsp/BORA micelles under thrombosis-associated oxidative conditions. Red downward arrows indicate the proposed reductions in platelet count, inflammatory cytokine levels, and H_2_O_2_ levels.

Herein, we developed a P2-functionalized and H_2_O_2_-responsive polymeric micelle for thrombus microenvironment modulation. A candidate fibrin-binding peptide, P2 (VTFIKC), was first screened using computer-aided drug design (CADD), and its fibrin-binding and thrombus-association properties were supported by in vitro assays. An atRA-based boronate ester prodrug, termed BORA, was then synthesized by conjugating atRA to an H_2_O_2_-responsive boronate linker, with the aim of enabling H_2_O_2_-dependent cleavage and the release of atRA and p-hydroxybenzyl alcohol (HBA) under oxidative conditions. The peptide-modified amphiphilic polymer P2-Mal-PEG-b-PAsp was further constructed and co-assembled with BORA to form P2-Mal-PEG-b-PAsp/BORA micelles. The micelles were designed to combine P2-mediated fibrin-binding potential with H_2_O_2_-responsive release and H_2_O_2_ scavenging, thereby modulating oxidative, inflammatory, and platelet-related changes associated with thrombosis (Figure 1). The physicochemical properties, H_2_O_2_ responsiveness, in vitro cytoprotective and anti-inflammatory effects, together with preliminary in vivo effects of the micelles in a thrombosis model, were systematically evaluated.

## 2. Materials and Methods

### 2.1. Chemical Reagents

Tetrahydrofuran (THF) and Cyanine 5.5 (Cy5.5) was purchased from Shanghai Aladdin Bio-Chem Technology Co., Ltd. (Shanghai, China). *n*-Hexane, *N*,*N*-dimethylformamide (DMF), anhydrous diethyl ether, anhydrous ethanol, dichloromethane, ethyl acetate, methanol, dichloroethane, anhydrous sodium sulfate, hydrogen peroxide (H_2_O_2_), calcium chloride (CaCl_2_), acetonitrile, phosphotungstic acid, potassium bromide, concentrated nitric acid, and acetone were purchased from Sinopharm Chemical Reagent Co., Ltd. (Shanghai, China). Triphosgene, benzyl-L-aspartate, all-trans retinoic acid, 4-(bromomethyl)phenylboronic acid, trimethylolethane, cesium carbonate, dichloroacetic acid, hydrogen bromide/acetic acid solution, and deuterated dimethyl sulfoxide were obtained from Shanghai Bide Pharmaceutical Technology Co., Ltd. (Shanghai, China). Dimethyl sulfoxide (DMSO) for polymer synthesis was purchased from Sinopharm Chemical Reagent Co., Ltd., while DMSO used for cell experiments was obtained from Nanjing Chemical Reagent Co., Ltd. (Nanjing, China). Thrombin and Tween-20 were purchased from Sigma-Aldrich, Shanghai, China. Maleimide-poly(ethylene glycol)-amine (Mal-PEG-NH_2_) was purchased from Xi’an Ruixi Biological Technology Co., Ltd. (Xi’an, China). The retinoic acid boronate ester prodrug BORA, Mal-PEG-b-PAsp, P2-Mal-PEG-b-PAsp, blank P2-Mal-PEG-b-PAsp micelles, and BORA-loaded P2-Mal-PEG-b-PAsp micelles were synthesized or prepared in-house as described in the experimental procedures.

### 2.2. Biological and Cell Culture Reagents

Fibrin, fibrinogen, and D-dimer were purchased from Sigma-Aldrich, Shanghai, China. The Monolith RED-NHS protein labelling kit was obtained from NanoTemper Technologies, Munich, Germany. Phosphate-buffered saline (PBS, pH 7.4), DMEM medium, penicillin-streptomycin, the reactive oxygen species (ROS) detection kit, and PBS buffer (pH 7.4) were obtained from Nanjing KeyGen Biotech Co., Ltd., Nanjing, China. Fetal bovine serum (FBS) was purchased from Gibco, Waltham, MA, USA. Trypsin was obtained from Nanjing Wisent Biotechnology Co., Ltd., Nanjing, China. Peptides designed by our research group including MRFIEC, VTFIKC, MRFVKS, MGFIKG, and MTLVKS were synthesized by Sangon Biotech Co., Ltd., Shanghai, China. The CCK-8 assay kit was purchased from Beyotime Biotechnology Co., Ltd., Shanghai, China. TNF-α and IL-1β ELISA kits were purchased from R&D Systems, Minneapolis, MN, USA. Ferric chloride hexahydrate (FeCl_3_·H_2_O, AR) and chloral hydrate were purchased from Shanghai Aladdin Bio-Chem Technology Co., Ltd. Paraformaldehyde was obtained from iFangBio, Changsha, China. Heparin sodium anticoagulant tubes and EDTA anticoagulant tubes were purchased from Jiangsu Kangjian Medical Apparatus Co., Ltd., Taizhou, China.

### 2.3. Cell Lines

Human umbilical vein endothelial cells (HUVECs) were obtained from the Shanghai Institutes for Biological Sciences, Chinese Academy of Sciences. Cells were cultured at 37 °C in a humidified atmosphere containing 5% CO_2_ using DMEM complete medium supplemented with 10% FBS.

### 2.4. Animals and Ethical Approval

Female Sprague–Dawley (SD) rats aged 6 to 8 weeks with body weights ranging from 180 to 220 g were purchased from Jiangsu Qinglongshan Biotechnology Co., Ltd. (Zhenjiang, China, License No.: SCXK (Su) 2024-0001) and were used consistently throughout this preliminary study to reduce sex-related biological heterogeneity within the limited cohort and to maintain consistency across experimental groups. Fresh rat whole blood, plasma, and red blood cells were used for the in vitro thrombus adhesion and hemolysis assays. The rats were reared in specific pathogen-free (SPF) animal rooms using individual ventilated cages (IVC). The housing conditions were set at a temperature of (22 ± 2) °C, relative humidity of (50 ± 10)%, and a 12 h light/dark cycle, with free access to standard diet and drinking water. All animal experiments were performed in strict accordance with the Laboratory Animal Management Regulations of China Pharmaceutical University and approved by the Animal Ethics Committee of China Pharmaceutical University (Ethics Approval No.: 2025-03-002; Date: 3rd March 2025). The 3R principles (Reduction, Replacement, Refinement) were fully implemented throughout the experiments to minimize animal suffering. Carbon dioxide inhalation was adopted for euthanasia in compliance with ethical standards.

### 2.5. Computer-Aided Design and Screening of Fibrin-Binding Peptides

The crystal structure of fibrin was obtained from the RCSB Protein Data Bank (PDB ID: 1FZC) and imported into Schrödinger 2021. The structure was prepared using the Protein Preparation Wizard by adding hydrogens, removing water molecules, assigning protonation states, completing missing side chains, optimizing the hydrogen-bond network, and performing energy minimization with the OPLS3 force field. Potential binding pockets were predicted using the SiteMap (2021-3) module and evaluated based on pocket size, SiteScore, and hydrophilic/hydrophobic characteristics. The optimized fibrin structure was also analysed using the FT-map server to identify binding hot spots and high-frequency residues involved in hydrogen-bonding and non-bonded interactions. Based on the combined SiteMap and FT-map results, the major binding region and key interacting residues were selected for peptide library construction and molecular docking.

A peptide library was constructed according to the Mekler–Idlis complementary peptide theory. Key residues in the selected fibrin-binding region were used to design sense peptide sequences, and the corresponding antisense peptide library was generated using Python 3.5. The peptide library was then imported into Schrödinger 2021 for docking-based screening. Peptide docking was performed using the Peptide Docking workflow, with the selected fibrin-binding pocket defined as the docking region. Binding conformations and interaction patterns were evaluated by Glide docking, followed by MM-GBSA calculations to estimate peptide–fibrin binding affinity. Candidate peptides were ranked based on docking score, predicted binding energy, binding orientation, and interactions with key residues, including hydrogen-bonding, hydrophobic, and π–π interactions. Five hexapeptides were selected for further experimental validation: MRFIEC (P1), VTFIKC (P2), MRFVKS (P3), MGFIKG (P4), and MTLVKS (P5).

### 2.6. Stability Evaluation of Candidate Peptides

The chemical stability of the candidate peptides was evaluated under different stress conditions, including acidic, alkaline, high-temperature, and repeated freeze–thaw treatments. Briefly, peptide solutions were subjected to acidic conditions (pH 2), alkaline conditions (pH 12), high-temperature treatment (50 °C), or repeated freeze–thaw cycles. Untreated peptide solutions were used as controls. After treatment, the remaining peptide content was analysed by high-performance liquid chromatography (HPLC). Peptide degradation was evaluated by comparing the peak area of each treated sample with that of the untreated control at the same retention time.

HPLC analysis was performed using a mobile phase consisting of acetonitrile and water (10:90, *v*/*v*). The column temperature was maintained at 25 °C, the detection wavelength was set at 214 nm, and the flow rate was 1 mL/min.

### 2.7. In Vitro Fibrin-Binding and Thrombus-Association Assays

The binding affinity of the candidate peptides toward fibrin was evaluated by microscale thermophoresis (MST). Before MST analysis, fibrin was fluorescently labelled using a Monolith RED-NHS protein labelling kit. The buffer-exchange column was placed in a 1.5 mL centrifuge tube and centrifuged at 1500× *g* for 1 min to remove the storage solution. NHS buffer (300 μL) was added to the column and centrifuged at 1500× *g* for 1 min; this washing step was repeated three times. The target protein solution was then loaded onto the column and centrifuged at 1500× *g* for 2 min to collect the buffer-exchanged protein. RED-NHS dye was dissolved in DMSO to 600 μM and diluted with NHS labelling buffer to 300 μM. Subsequently, 90 μL of buffer-exchanged protein solution and 10 μL of dye solution were mixed in a 1.5 mL centrifuge tube and incubated in the dark for 30 min. After labelling, the mixture was transferred to an equilibrated purification column, and 550 μL of binding reaction buffer was added to collect the fluorescently labelled protein.

For MST analysis, peptide solutions were serially diluted with PBS containing 0.05% Tween-20 over a concentration range of 1 mM to 50 μM. Equal volumes of peptide solution and fluorescently labelled protein solution were mixed in PCR tubes and loaded into MST capillaries. Binding affinity was measured using a Monolith NT.115 instrument according to the manufacturer’s instructions, and the dissociation constant (*K*_d_) was calculated. Fibrinogen and D-dimer were used as control proteins to assess binding specificity. The *K*_d_ and specificity values were obtained from three independent measurements and expressed as mean ± standard deviation. The specificity was calculated according to the following equation:

$$Specificity=\frac{K_{d,fibrinogen}}{K_{d,fibrin}}$$

The association of the candidate peptides with in vitro-formed thrombi was evaluated using a thrombus adhesion assay. Fresh rat blood was collected by cardiac puncture into heparin sodium anticoagulant tubes and centrifuged at 2500× *g* for 10 min at 4 °C to obtain plasma. Thrombus formation was induced by adding thrombin (1 U) and CaCl_2_ (20 mM) to the plasma. After formation, the thrombi were washed three times with PBS to remove residual soluble components. Fluorescently labelled peptides were added to the thrombus samples and incubated for 30 min. The thrombi were then washed three times with PBS to remove unbound peptides. Fluorescence signals were detected using an in vivo imaging system, and fluorescence intensity was quantified and normalized to thrombus area to compare thrombus association among different candidate peptides.

### 2.8. Protection Against H_2_O_2_-Induced Endothelial Injury

The protective effect of micelles against H_2_O_2_-induced endothelial injury was evaluated using a CCK-8 assay. HUVECs in the logarithmic growth phase were digested, resuspended in complete DMEM medium, counted using a hemocytometer, and seeded into 96-well plates at 10,000 cells per well in 100 μL medium. After incubation at 37 °C in a humidified 5% CO_2_ atmosphere for 24 h, the culture medium was removed.

Cells were treated with free HBA (30 μg/mL), free atRA (60 μg/mL), HBA plus atRA at the same concentrations, or BORA-loaded P2-Mal-PEG-b-PAsp micelles (100 and 200 μg/mL). After 4 h of incubation, 100 μL of H_2_O_2_ solution (200 μM) was added to each well, followed by further incubation for 24 h. Untreated cells served as the normal control, and cells treated with H_2_O_2_ alone served as the oxidative injury model group.

After H_2_O_2_ stimulation, 20 μL of CCK-8 solution was added to each well and incubated for 4 h at 37 °C. After shaking for 10 min, absorbance was measured at 450 nm using a microplate reader. Cell viability was calculated relative to the negative control group, and the cytoprotective effect was evaluated by comparing treated groups with the H_2_O_2_ model group.

### 2.9. Synthesis and Characterization of Components for Micelle Construction

**Figure 2:**
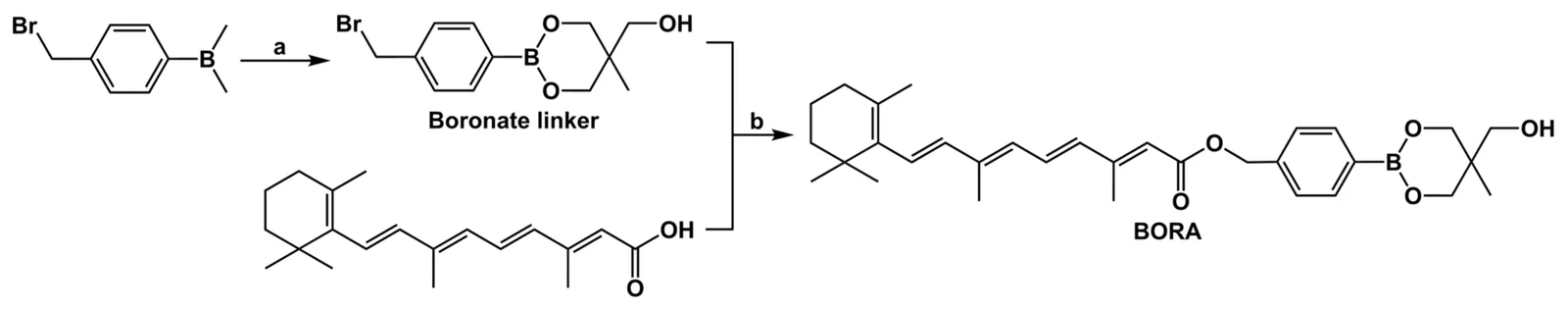
Synthesis of BORA: (a) Trimethylolethane, THF, rt, 4 h. (b) Cs_2_CO_3_, DMF, 40 °C, 24 h.

**Figure 3:**
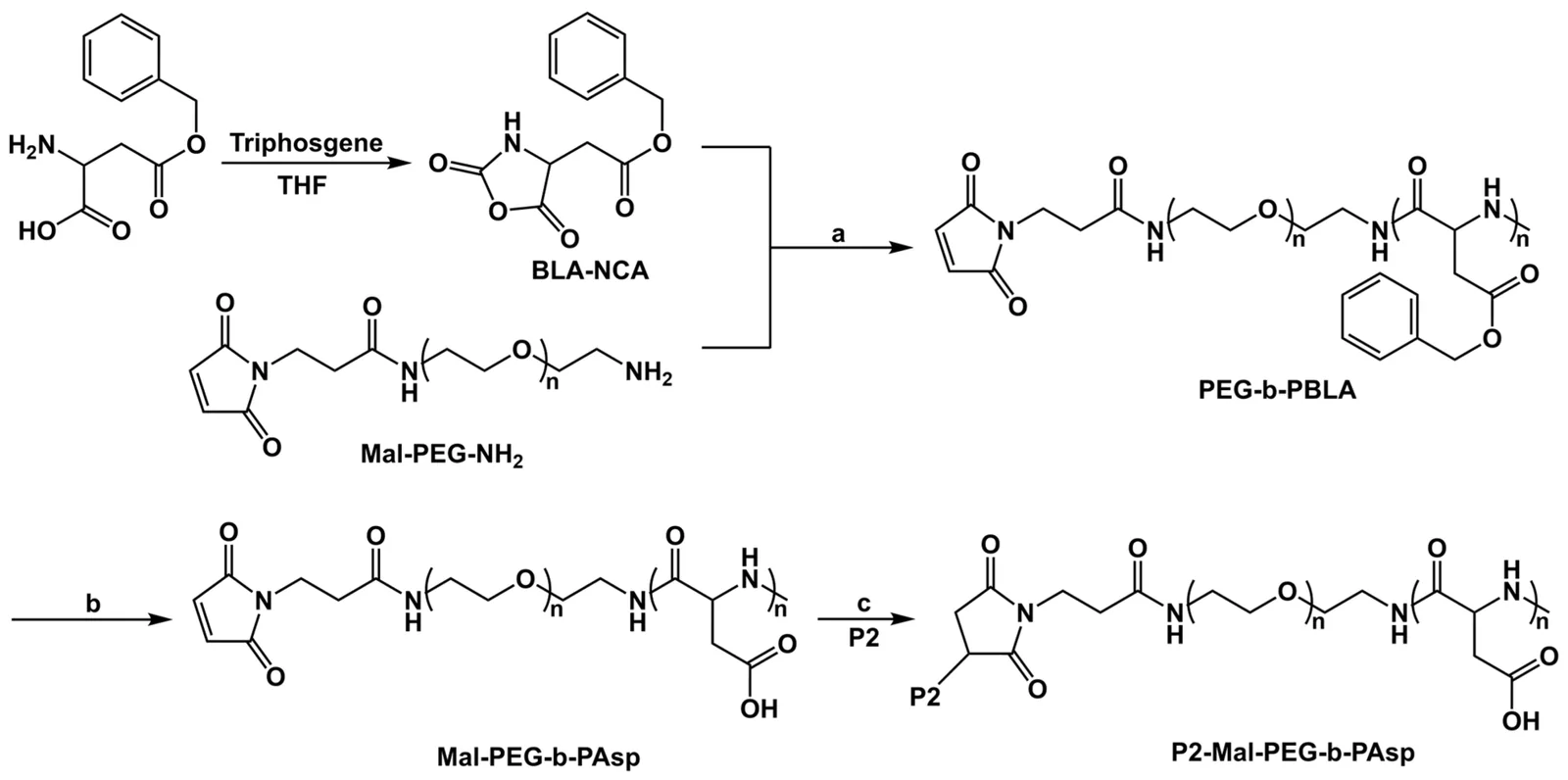
Synthesis of P2-Mal-PEG-b-PAsp (a) anhydrous DCM/DMF (3:1, *v*/*v*), N_2_, 40 °C, 72 h. (b) 33% HBr/AcOH, Cl_2_CHCOOH, rt, 6 h. (c) DMF, rt, 24 h.

The synthetic routes of BORA, Mal-PEG-b-PAsp, and P2-Mal-PEG-b-PAsp are shown in Figure 2 and Figure 3, and detailed synthetic procedures and characterization data are provided in the Supporting Information.

Briefly, a boronate ester-containing intermediate, hereafter referred to as the boronate linker, was first prepared from 4-(bromomethyl)phenylboronic acid and trimethylolethane. The boronate linker was subsequently reacted with all-trans retinoic acid (atRA) in the presence of cesium carbonate to afford the H_2_O_2_-responsive prodrug BORA. After extraction and chromatographic purification, the structure of BORA was confirmed by ^1^H NMR and ESI-MS.

Mal-PEG-b-PAsp was synthesized through N-carboxyanhydride ring-opening polymerization. β-Benzyl-L-aspartate N-carboxyanhydride (BLA-NCA) was first prepared from benzyl-L-aspartate and triphosgene and then polymerized using Mal-PEG-NH_2_ as the macroinitiator to obtain Mal-PEG-b-PBLA. The benzyl protecting groups were subsequently removed by acidolysis to afford Mal-PEG-b-PAsp. Polymers with different block ratios were prepared by adjusting the feed molar ratio of Mal-PEG-NH_2_ to BLA-NCA. The polymer structures and block ratios were characterized by ^1^H NMR.

Using P2 as the selected candidate fibrin-binding peptide, P2-Mal-PEG-b-PAsp was synthesized through a thiol-maleimide Michael addition reaction between the terminal cysteine residue of P2 and the maleimide group of Mal-PEG-b-PAsp. After the reaction, the product was purified by dialysis against deionized water and collected by lyophilization. Successful conjugation of P2 to Mal-PEG-b-PAsp was confirmed by the disappearance of the maleimide proton signal in the ^1^H NMR spectrum and by gel permeation chromatography (GPC).

### 2.10. Preparation of P2-Mal-PEG-b-PAsp/BORA Micelles

Blank and BORA-loaded P2-Mal-PEG-b-PAsp micelles were prepared using a solvent exchange method. For the preparation of blank micelles, P2-Mal-PEG-b-PAsp polymer (10 mg) was dissolved in DMF (5 mL) under magnetic stirring. Subsequently, ultrapure water (10 mL) was slowly added dropwise to the polymer solution, and the mixture was stirred at room temperature for 2 h. The resulting solution was transferred into a dialysis bag and dialyzed against ultrapure water for 24 h to remove DMF, yielding blank P2-Mal-PEG-b-PAsp micelles. For the preparation of BORA-loaded micelles, P2-Mal-PEG-b-PAsp polymer (10 mg) and BORA prodrug were co-dissolved in DMF (5 mL) under magnetic stirring. Ultrapure water (10 mL) was then slowly added dropwise to the mixed solution, followed by stirring at room temperature for 2 h to allow micelle self-assembly. The solution was subsequently transferred into a dialysis bag and dialyzed against ultrapure water for 24 h to remove DMF and unencapsulated components, affording BORA-loaded P2-Mal-PEG-b-PAsp micelles.

### 2.11. Characterizations of P2-Mal-PEG-b-PAsp/BORA Micelles

After dialysis, blank P2-Mal-PEG-b-PAsp micelles and BORA-loaded P2-Mal-PEG-b-PAsp micelles were characterized for hydrodynamic diameter, particle size distribution, polydispersity index (PDI), and zeta potential by dynamic light scattering (DLS). Before measurement, the micelle suspensions were appropriately diluted with ultrapure water to avoid multiple scattering. Each sample was equilibrated at room temperature and measured in triplicate, and results were expressed as mean ± standard deviation. The triplicate measurements represented repeated instrument readings of the same sample rather than independently prepared formulation batches.

The morphology of BORA-loaded micelles was observed by transmission electron microscopy (TEM). Briefly, a drop of diluted micelle suspension was placed onto a carbon-coated copper grid and allowed to adsorb for several minutes. Excess liquid was removed with filter paper, followed by negative staining with phosphotungstic acid solution. After air-drying at room temperature, the samples were observed by TEM to evaluate micellar morphology and nanoscale structure.

The BORA loading content was determined by inductively coupled plasma-based boron quantification. A known amount of lyophilized BORA-loaded micelles was digested with concentrated nitric acid until a clear solution was obtained. The digest was diluted to a fixed volume with ultrapure water, and the boron concentration was quantified using a standard calibration curve. Since each BORA molecule contains one boron atom, the amount of BORA incorporated into the micelles was calculated from the measured boron content and the theoretical boron content of BORA. Blank micelles were processed in the same manner and used as background controls. The denominator was the total mass of the lyophilized BORA-loaded micellar formulation, including both polymer and BORA. The apparent drug-loading content was calculated according to the following equation. The reported value was derived from one independently prepared micelle batch; recovery, encapsulation efficiency, and batch-to-batch variability were not independently determined. Drug loading content was calculated according to the following equation:

$$Drug\;loading\;content\;(\%)=\frac{W_{BOR\mathrm{A-l}oaded}}{W_{micelles}}\times 100$$

### 2.12. H_2_O_2_ Responsiveness Assay

BORA-loaded micelles were prepared as described above and transferred into a dialysis bag. The dialysis bag was immersed in PBS containing 1 mM H_2_O_2_ and incubated at 37 °C in a shaking incubator for 4 h. After incubation, the micelle solution was collected, and the hydrodynamic diameter and polydispersity index were measured by dynamic light scattering (DLS) to assess H_2_O_2_-induced micellar structural changes.

### 2.13. In Vitro Drug Release Assay

P2-Mal-PEG-b-PAsp polymer and BORA prodrug were dissolved in DMF and slowly added dropwise into water under stirring to allow micelle formation. The resulting micelle solution was transferred into a dialysis bag with a molecular weight cut-off of 3000 Da. PBS containing 200 μM H_2_O_2_ (pH 7.4) was used as the external release medium to simulate an oxidative physiological environment. The dialysis system was incubated at 37 °C in a shaking incubator. At 1, 2, 4, 6, 10, 12, 24, and 48 h after initiation of the release assay, 1 mL of the external medium was collected and replaced with an equal volume of fresh release medium to maintain a constant total volume of 60 mL. The absorbance of released all-trans retinoic acid was measured at 350 nm using a UV–vis spectrophotometer. A standard curve of atRA was established by UV–vis spectrophotometry, and the cumulative release profile was calculated accordingly. As UV–vis measurement at this wavelength may not unambiguously distinguish free atRA from intact BORA or other retinoid-containing species, the results were expressed as apparent atRA-equivalent release rather than definitive release of free atRA, and used only as a simple and preliminary reference for monitoring changes associated with the H_2_O_2_-triggered reaction.

### 2.14. H_2_O_2_ Scavenging Assay

The H_2_O_2_-scavenging capacity of the micelles was determined using a commercial H_2_O_2_ detection kit based on titanium sulfate colorimetry, a method previously applied to evaluate H_2_O_2_ consumption by nanomaterial systems [32,33]. BORA-loaded micelles at different concentrations (200, 400, and 600 μg/mL, 200 μL) were added to H_2_O_2_ solution (200 μM) and incubated at 37 °C for 2 h. After incubation, the titanium sulfate reagent and alkaline solution from the assay kit were added, and the mixture was allowed to stand at room temperature for 5 min. The samples were then centrifuged at 500× *g* for 10 min, and the supernatant was discarded. The resulting precipitate was washed twice with cold acetone and dissolved for absorbance measurement at 412 nm using a microplate reader. Free atRA and HBA, the degradation products of BORA after ROS responsiveness, were used as control groups. The H_2_O_2_-scavenging efficiency was calculated from the residual H_2_O_2_-associated absorbance.

### 2.15. Hemocompatibility Assays

Fresh rat whole blood was collected into heparin sodium anticoagulant tubes and centrifuged at 300× *g* for 20 min at room temperature. After centrifugation, the supernatant was discarded, and the red blood cells were washed several times with PBS. The purified red blood cells were then diluted to 10% with normal saline. Subsequently, 100 μL of the red blood cell suspension was transferred into 1.5 mL centrifuge tubes and incubated with peptide samples at different concentrations (50, 100, and 200 μg/mL). Deionized water and PBS were used as the positive and negative controls, respectively. After incubation at 37 °C for 4 h, the samples were centrifuged at 300× *g* for 5 min. The supernatant was collected, and the absorbance at 541 nm was measured using a microplate reader. The hemolysis rate was calculated according to the following equation:

$$Hemolysis\;rate\;\left(\%\right)=\frac{A_{sample}\;-\;A_{negative\;control}}{A_{positive\;control}\;-\;A_{negative\;control}}\times 100\%$$

### 2.16. Cytocompatibility Assays

HUVECs in the logarithmic growth phase were digested, resuspended in complete culture medium, and counted using a hemocytometer. The cells were seeded into 96-well plates at a density of 5000 cells per well and incubated at 37 °C in a humidified atmosphere containing 5% CO_2_ for 24 h. The culture medium was then replaced with fresh medium containing different concentrations of candidate peptides (67.5, 125, 250, 500, and 1000 μg/mL), followed by further incubation for 72 h. After incubation, 20 μL of CCK-8 solution was added to each well, and the cells were incubated for another 4 h. The absorbance at 450 nm was measured using a microplate reader. Cell viability was calculated using the following equation:

$$Cell\;viability\;(\%)\;=\frac{{OD}_{treated}\;-\;{OD}_{blank}}{{OD}_{control}\;-\;{OD}_{blank}}\times 100\%$$

The cytocompatibility of BORA-loaded P2-Mal-PEG-b-PAsp micelles was further assessed in HUVECs using a CCK-8 assay. HUVECs were seeded into 96-well plates and incubated overnight to allow cell attachment. The cells were then treated with BORA-loaded micelles at different concentrations and incubated under standard cell culture conditions. After treatment, CCK-8 solution was added to each well and incubated to allow formazan formation. The absorbance was measured using a microplate reader, and cell viability was calculated relative to the untreated control group. The assay was used to evaluate the concentration-dependent cytotoxicity of the micelles and to determine a suitable concentration range for subsequent in vitro efficacy studies.

### 2.17. Intracellular ROS Detection in HUVECs

Intracellular ROS levels were detected using DCFH-DA as a fluorescent probe. HUVECs were seeded into confocal dishes at 1 × 10^5^ cells per dish and incubated at 37 °C in a humidified 5% CO_2_ atmosphere for 24 h. After cell attachment, the medium was removed, and cells were treated with free atRA, free HBA, or BORA-loaded P2-Mal-PEG-b-PAsp micelles for 3 h. H_2_O_2_ was then added at a final concentration of 200 μM, followed by incubation for 24 h to induce intracellular oxidative stress.

After treatment, the medium was discarded, and cells were washed three times with PBS. DCFH-DA solution (10 μM, 1 mL) was added to each confocal dish and incubated at 37 °C for 20 min. The DCFH-DA solution was then removed, and cells were washed with PBS to eliminate extracellular probe. Finally, 200 μL of PBS was added to each dish, and samples were protected from light before imaging. Green DCF fluorescence was visualized using an LSM 800 confocal laser scanning microscope (Carl Zeiss Microscopy GmbH, Jena, Germany), and fluorescence intensity was used to compare intracellular ROS accumulation among different groups.

### 2.18. Anti-Inflammatory Activity in HUVECs

The anti-inflammatory activity of BORA-loaded P2-Mal-PEG-b-PAsp micelles was evaluated by measuring TNF-α and IL-1β levels in H_2_O_2_-stimulated HUVECs using ELISA. HUVECs were seeded into 6-well plates at 3 × 10^5^ cells per well and incubated at 37 °C in a humidified 5% CO_2_ atmosphere for 24 h. After cell attachment, the medium was removed, and cells were treated with free HBA, free atRA, HBA plus atRA, or BORA-loaded P2-Mal-PEG-b-PAsp micelles for 3 h. H_2_O_2_ was then added at a concentration of 200 μM, followed by incubation for 24 h to induce inflammatory activation. Untreated cells served as the normal control, and cells treated with H_2_O_2_ alone served as the inflammatory model group.

After treatment, culture supernatants were collected and centrifuged at 100× *g* for 5 min to remove cell debris. TNF-α and IL-1β levels in the supernatants were quantified using the corresponding ELISA kits according to the manufacturers’ instructions. Standards and samples were added to ELISA plates, with duplicate wells for each standard and triplicate wells for each sample. Absorbance was measured using a microplate reader, and cytokine concentrations were calculated from the corresponding standard curves. The anti-inflammatory activity of each formulation was evaluated by comparing TNF-α and IL-1β levels among different treatment groups.

The reported n values for cell viability, intracellular ROS and ELISA assays represent independent biological experiments.

### 2.19. Preliminary In Vivo Evaluation in FeCl_3_-Induced Thrombosis

The preliminary in vivo effects of BORA-loaded P2-Mal-PEG-b-PAsp micelles were evaluated in a FeCl_3_-induced carotid artery thrombosis model in female SD rats. Rats were anesthetized by intraperitoneal injection of 10% chloral hydrate at 40 mg/kg, fixed in the supine position, and shaved in the neck region. A longitudinal incision was made along the neck midline, and the surrounding muscles were bluntly separated with hemostatic forceps to expose the left carotid artery. Body temperature was maintained at 37 °C using a heating pad during surgery.

To induce thrombosis, filter paper (1 cm × 2 cm) soaked in 10% FeCl_3_ aqueous solution was wrapped around the exposed left carotid artery for 5 min and covered with plastic film to prevent nonspecific injury to adjacent tissues. After FeCl_3_ treatment, the plastic film and filter paper were removed, and the injured vascular area was gently rinsed with PBS to remove residual FeCl_3_.

To evaluate the ex vivo biodistribution of the micelles, Cy5.5 was physically encapsulated within the hydrophobic micellar core during micelle preparation. Cy5.5-loaded micelles without P2 and Cy5.5-loaded complete P2-functionalized micelles were administered via the tail vein at an equivalent Cy5.5 dose of 100 μg/kg. At 2 h [17] after administration and following blood collection, the rats were perfused with PBS through the cardiac apex. The heart, liver, spleen, lungs, kidneys, contralateral carotid artery, and thrombotic carotid artery were then excised and subjected to ex vivo fluorescence imaging. Relative fluorescence intensity was quantified using the analysis software supplied with the fluorescence imaging system.

For the evaluation of in vivo antithrombotic effects, different formulations were administered immediately via tail vein injection. The blank group received normal saline without FeCl_3_ treatment, while the model group received normal saline after FeCl_3_-induced thrombosis. Treatment groups received free atRA, free HBA, atRA plus HBA, drug-free micelles, micelles without P2 or BORA-loaded P2-Mal-PEG-b-PAsp micelles at 10 mg/kg in an injection volume of 200 μL. Animals were randomly assigned to the experimental groups. No animals or measurements were excluded from the final analysis. Formal allocation concealment and investigator blinding were not implemented during treatment administration or outcome assessment.

At 2 h after administration, whole blood was collected by cardiac puncture into EDTA anticoagulant tubes. The tubes were gently inverted to mix the blood with anticoagulant and stored at 4 °C before analysis. Platelet count, mean platelet volume, white blood cell count, and lymphocyte count were measured using an automated hematology analyzer.

The reported n values in this experiment correspond to independent biological replicates.

### 2.20. Statistical Analysis

All statistical analyses were performed using R software (version 4.6.1). Comparisons involving multiple treatment groups or dose levels were analyzed using one-way or two-way analysis of variance (ANOVA), as appropriate for the experimental design, followed by multiplicity-adjusted post hoc comparisons. Unpaired two-tailed Student’s *t*-tests were retained only for direct comparisons between two independent groups. Data are presented as mean ± standard deviation (SD). Statistical significance was determined using multiplicity-adjusted *p* values where applicable: ns, adjusted *p* > 0.05; * adjusted *p* < 0.05; ** adjusted *p* < 0.01; *** adjusted *p* < 0.001; and **** adjusted *p* < 0.0001.

## 3. Results and Discussion

### 3.1. Selection and In Vitro Evaluation of a Candidate Fibrin-Binding Peptide

**Figure 4:**
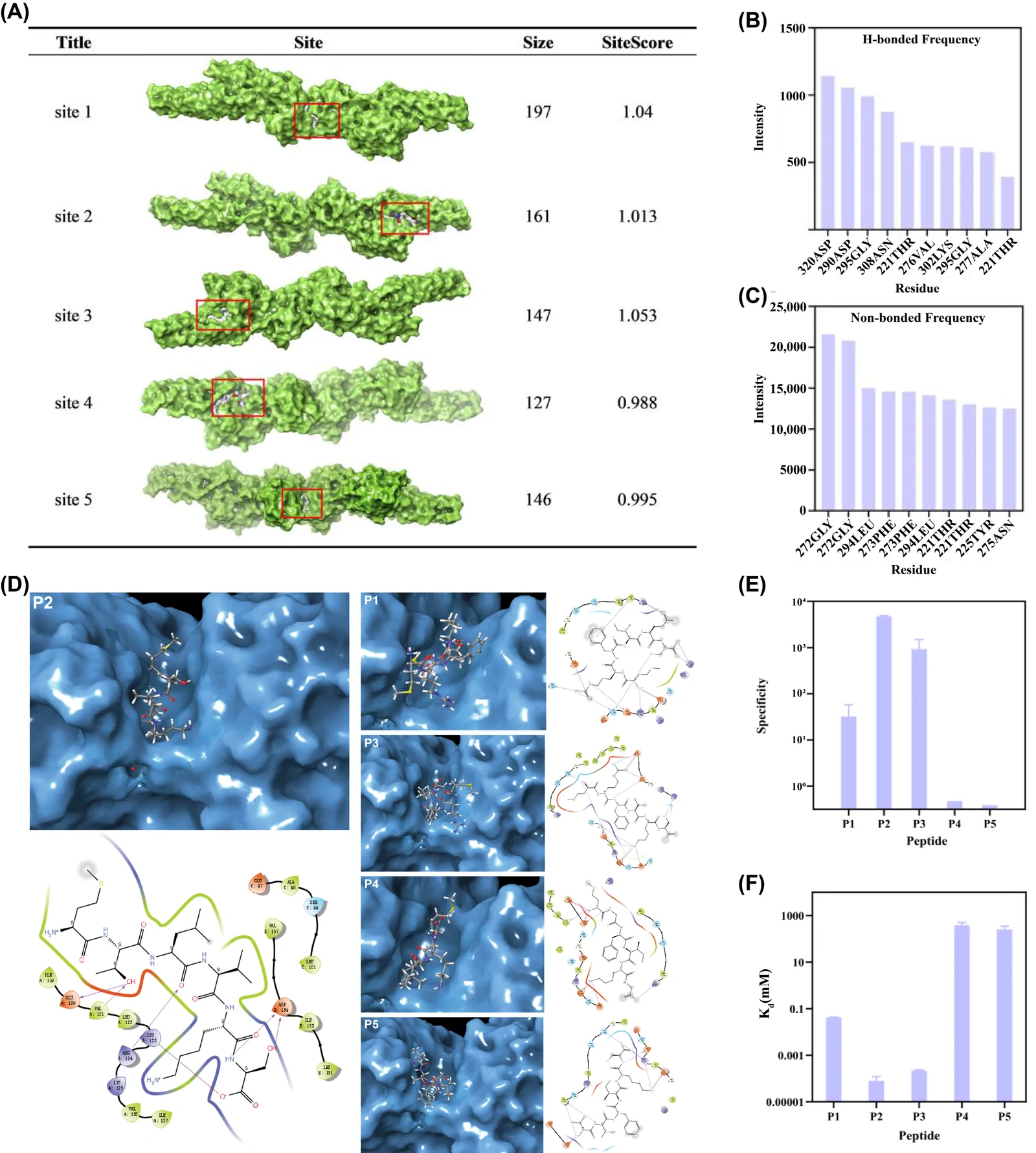
Computational screening and fibrin-binding evaluation of candidate peptides. (**A**) Prediction of five potential fibrin-binding pockets using Schrödinger SiteMap, with the pocket size and SiteScore shown for each candidate site. (**B**,**C**) FT-map analysis of residues with high frequencies of hydrogen-bonded interactions (**B**) and nonbonded interactions (**C**) within the selected binding region. (**D**) Predicted binding poses and two-dimensional interaction diagrams of candidate peptides P1–P5 within the fibrin-binding pocket; the binding mode of P2 is shown at higher magnification. In (**D**), the blue molecular surface represents fibrin, while green, orange, purple, and light blue in the two-dimensional diagrams denote hydrophobic, negatively charged, positively charged, and polar residues, respectively; purple arrows indicate hydrogen bonds (**E**,**F**) MST-derived binding specificity (**E**) and dissociation constants (*K*_d_) (**F**) of P1–P5 toward fibrin. Data are presented as mean ± SD.

CADD-based screening was first used to identify candidate fibrin-binding peptides. SiteMap analysis identified five potential binding pockets on fibrin, with site 1 selected for subsequent peptide docking based on the favorable combination of pocket size and SiteScore, and FT-map analysis further predicted key interacting residues within this selected region (Figure 4A–C). Five hexapeptides, designated P1–P5, were further selected as candidate fibrin-binding peptides after peptide docking and MM-GBSA evaluation (Figure 4D & Table S1), and the key interactions between the peptides and the receptor were predicted by molecular docking. In MST analysis, P1, P2, and P3 demonstrated generally lower dissociation constants and higher specificity than P4 and P5. Among the tested peptides, P2 showed the lowest apparent dissociation constant and the highest calculated specificity toward fibrin under the applied MST conditions (Figure 4E,F). Based on these results, P1, P2, and P3 were advanced to subsequent evaluations, with P2 showing the strongest overall fibrin-binding performance among the tested candidates.

**Figure 5:**
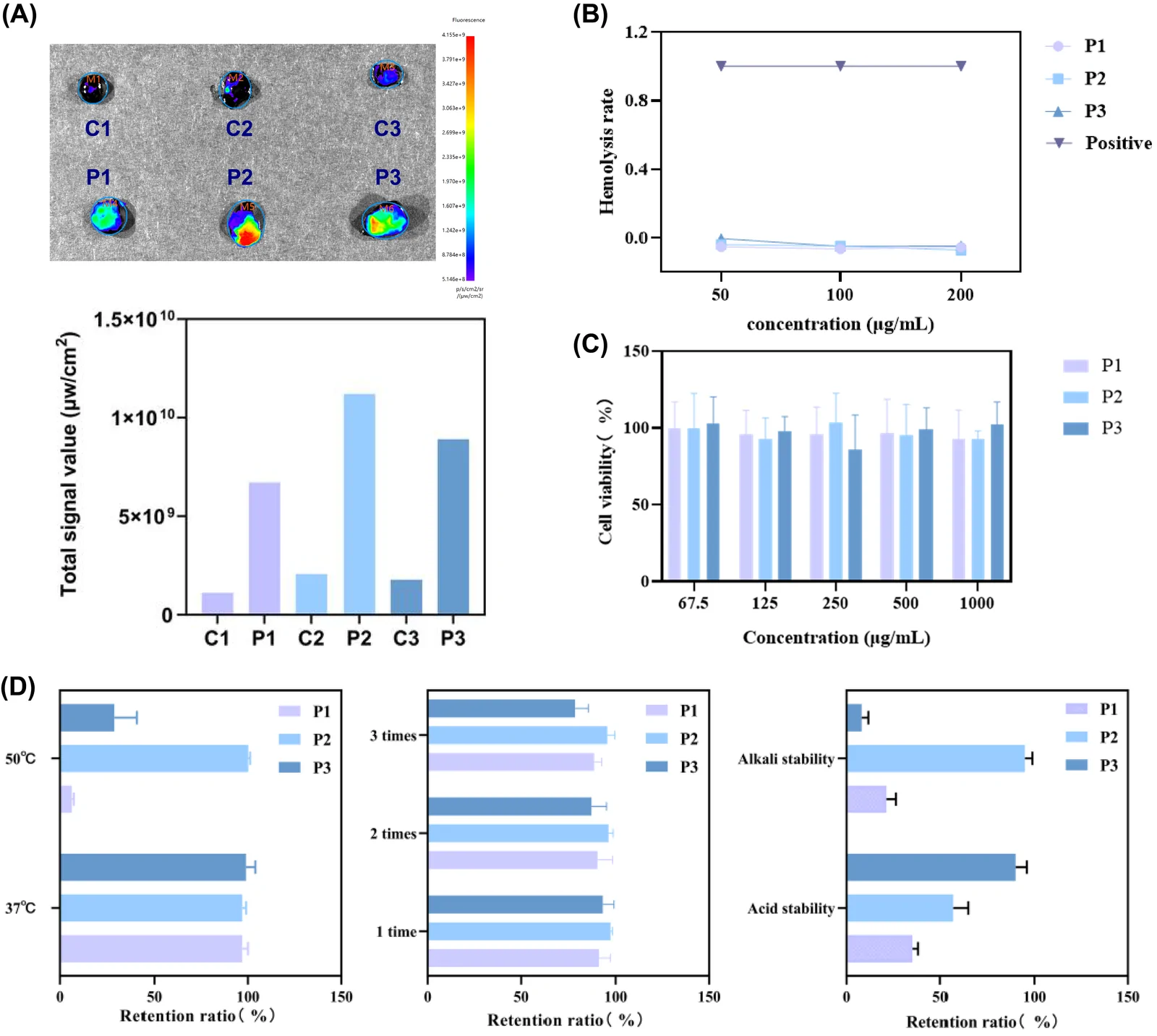
Experimental validation, biocompatibility, and stability of selected fibrin-binding peptides. (**A**) Representative fluorescence images of thrombi incubated with P1–P3 or the corresponding control peptides (C1–C3), together with quantitative analysis of the total fluorescence signal normalized to thrombus area. (**B**) Hemolysis rates of P1–P3 at different concentrations; deionized water was used as the positive control. (**C**) Viability of HUVECs treated with different concentrations of P1–P3, as determined by the CCK-8 assay. (**D**) Retention ratios of P1–P3 after thermal treatment at 37 and 50 °C, repeated freeze–thaw cycles, and exposure to acidic or alkaline conditions. Data are presented as mean ± SD.

The thrombus-association capability of these three peptides was subsequently evaluated with an in vitro adhesion assay. Consistent with the result of the MST assay, P2 produced the strongest fluorescence signal in the thrombus model, whereas P1, P3, and the control peptide group exhibited weaker thrombus association (Figure 5A). The biocompatibility and stability of the candidate peptides were further assessed. Hemolysis assays showed negligible red blood cell lysis within the tested concentration range, indicating low hemolytic activity under the tested conditions (Figure 5B). CCK-8 assays in HUVECs showed no obvious cytotoxicity after peptide treatment (Figure 5C). In addition, peptide stability analysis under thermal, acidic, alkaline, and repeated freeze–thaw conditions showed that P2 maintained higher overall retention than the other candidates under several of the tested conditions (Figure 5D).

Taken together, P2 presented the most balanced profile among the tested candidates in terms of fibrin binding, in vitro thrombus association, hemolytic activity, cytocompatibility, and chemical stability. Therefore, P2 was selected as the ligand for constructing the P2-functionalized micellar system.

### 3.2. Construction and Characterization of P2-Functionalized H_2_O_2_-Responsive BORA-Loaded Micelles

**Figure 6:**
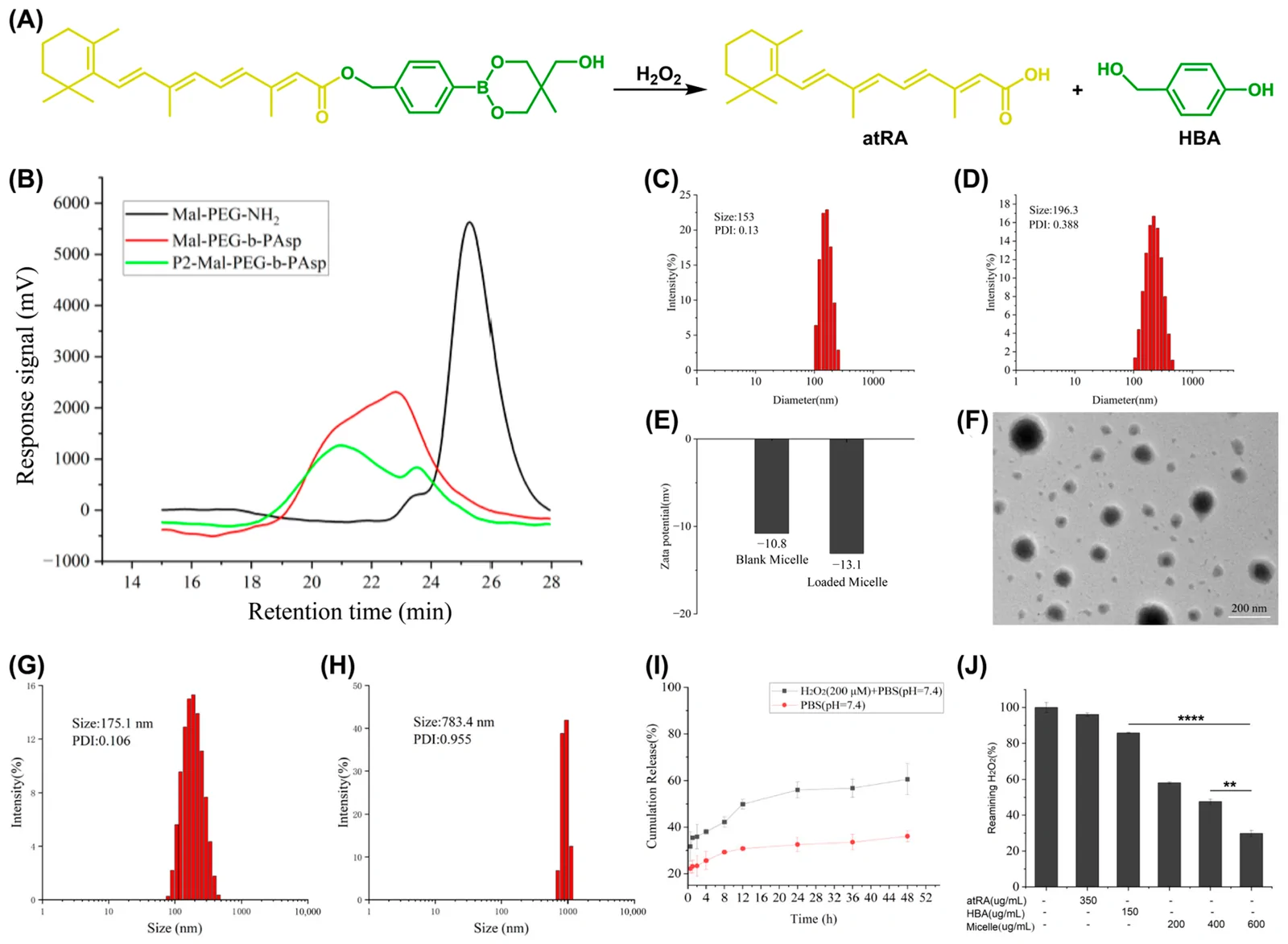
(**A**) Schematic illustration of H_2_O_2_-triggered BORA cleavage and atRA/HBA release. (**B**) GPC chromatograms of Mal-PEG-NH_2_, Mal-PEG-b-PAsp, and P2-Mal-PEG-b-PAsp. (**C**,**D**) DLS particle size distributions of blank P2-Mal-PEG-b-PAsp micelles and BORA-loaded P2-Mal-PEG-b-PAsp micelles, respectively. (**E**) Zeta potentials of blank and BORA-loaded micelles. (**F**) TEM image of BORA-loaded micelles. Scale bar: 200 nm. (**G**,**H**) Particle size distributions of BORA-loaded micelles before (**G**) and after (**H**) H_2_O_2_ treatment. (**I**) In vitro cumulative apparent atRA-equivalent release profiles of atRA from BORA-loaded micelles in PBS with or without H_2_O_2_. (**J**) H_2_O_2_-scavenging capacity of atRA, HBA, and BORA-loaded micelles at different concentrations. Data are presented as mean ± SD. ** *p* < 0.01; **** *p* < 0.0001.

In the designed micelles, BORA functioned as an H_2_O_2_-responsive prodrug that could undergo oxidative cleavage to release atRA and HBA, while P2-Mal-PEG-b-PAsp provided the amphiphilic micellar framework and potential P2-mediated fibrin-binding functionality (Figure 6A). The successful synthesis of BORA was confirmed by ^1^H NMR and ESI-MS (Figures S1 and S2). Mal-PEG-b-PAsp polymers with different block ratios were then obtained. As shown in Table S2, increasing the PAsp block length led to a more negative surface charge but a larger micelle size, while a shorter PAsp block length resulted in smaller micelles with a less negative zeta potential. Therefore, a moderate PAsp block length was expected to provide sufficient anionic character for colloidal stabilization while avoiding excessive particle enlargement after BORA loading, which could otherwise increase susceptibility to mononuclear phagocyte system clearance [34,35,36,37]. Mal-PEG45-b-PAsp15 provided an intermediate particle size and a moderately negative zeta potential, suggesting a practical balance between nanoscale dimensions and anionic character. Thus, Mal-PEG_45_-b-PAsp_15_ was selected as the polymer framework for constructing BORA-loaded P2-functionalized micelles. Then, P2 was conjugated to Mal-PEG-b-PAsp through thiol-maleimide coupling to obtain P2-Mal-PEG-b-PAsp. The disappearance of the maleimide proton signal at δ 8.20 ppm in the ^1^H NMR spectrum and the decreased GPC retention time supported the successful conjugation of P2 to the polymer backbone (Figure S3 and Figure 6B). Additional ^1^H NMR spectra of the synthetic intermediates and Mal-PEG_45_-b-PAsp polymers with different PAsp block lengths are provided in Figures S8–S13.

The resulting P2-Mal-PEG-b-PAsp was further assembled with BORA to form P2-functionalized BORA-loaded micelles. DLS analysis showed that BORA loading increased the hydrodynamic diameter from approximately 153.0 nm for blank micelles to 196.3 nm for BORA-loaded micelles (Figure 6C,D). The drug-loaded micelles retained a negative zeta potential (Figure 6E), and TEM imaging showed discrete spherical micelles with particle sizes mainly ranging from 100 to 200 nm (Figure 6F). Under the applied ICP-based calculation method, the formulation yielded an apparent drug-loading content of 60.45%.

### 3.3. H_2_O_2_-Triggered Drug Release and H_2_O_2_-Scavenging Performance

H_2_O_2_-mediated cleavage of the boronate ester in BORA is expected to generate atRA and HBA while simultaneously consuming H_2_O_2_. Consistent with the design, H_2_O_2_ treatment caused a marked change in micelle size distribution and dispersity in DLS analysis, indicating substantial H_2_O_2_-induced changes in particle size and dispersity that were consistent with micellar destabilization or structural rearrangement (Figure 6G,H). These changes are consistent with, though do not directly prove, the cleavage of the boronate ester linkage and the formation of the proposed degradation products [38]. Under non-oxidative conditions, the micelles exhibited a slower release profile, whereas the H_2_O_2_-containing medium produced a faster apparent atRA-equivalent signal, suggesting that the micellar formulation may limit premature release under the tested conditions. In contrast, the presence of H_2_O_2_ produced a faster apparent atRA-equivalent signal, which is consistent with H_2_O_2_-dependent changes in release behavior and micellar stability (Figure 6I). In addition to triggered release, the micelles exhibited concentration-dependent H_2_O_2_-scavenging activity, reaching approximately 50% scavenging at 200 μg/mL and approximately 70% at 600 μg/mL. This effect was stronger than that of free atRA or HBA, suggesting that the enhanced H_2_O_2_ consumption may be largely associated with oxidation of the boronate ester-containing BORA structure (Figure 6J).

### 3.4. In Vitro Cytoprotective and Anti-Inflammatory Effects

**Figure 7:**
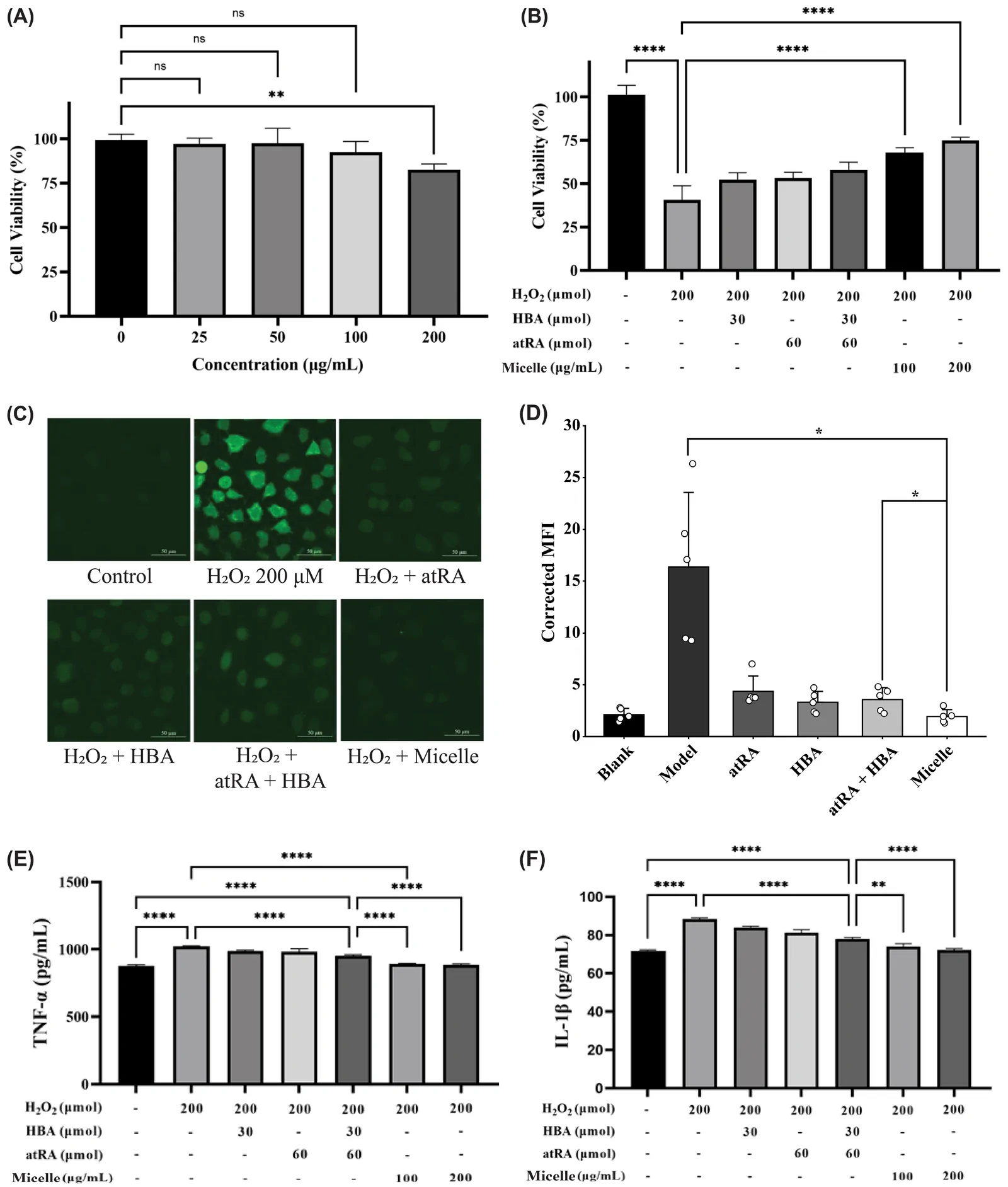
(**A**) Cytocompatibility of BORA-loaded micelles in HUVECs, n = 6. (**B**) Protective effects of atRA, HBA, atRA + HBA, and BORA-loaded micelles against H_2_O_2_-induced cytotoxicity in HUVECs (n = 6). (**C**) Intracellular ROS levels were detected by DCFH-DA staining after different treatments. Scale bar: 50 μm. (**D**) Semiquantitative analysis of the images in (**C**), expressed as background-corrected mean fluorescence intensity (corrected MFI, a.u.). (**E**,**F**) ELISA analysis of TNF-α (**E**) and IL-1β (**F**) levels in H_2_O_2_-stimulated HUVECs (n = 3). Data are presented as mean ± SD. ns, not significant; * *p* < 0.05; ** *p* < 0.01; **** *p* < 0.0001.

Using HUVECs as a simplified in vitro model, the cytocompatibility of P2-Mal-PEG-b-PAsp/BORA micelles was preliminarily assessed using a CCK-8 assay (Figure 7A). No obvious cytotoxicity was observed at concentrations below 100 μg/mL, and cell viability remained approximately 80% at 200 μg/mL, indicating acceptable cytocompatibility of the micelles.

The protective effect of the micelles was then assessed in an H_2_O_2_-induced HUVEC injury model (Figure 7B). H_2_O_2_ treatment reduced cell viability to approximately 40%, and BORA-loaded micelles increased cell viability to over 60% in a concentration-dependent manner, whereas free HBA and atRA showed only limited protective effects. These results suggest that the observed cytoprotective effect of P2-Mal-PEG-b-PAsp/BORA micelles may not be attributable solely to free atRA or HBA and may reflect the combined effects of BORA incorporation and micellar formulation.

To determine whether the improvement in cell viability was accompanied by attenuation of oxidative stress, intracellular ROS levels were examined using DCFH-DA staining (Figure 7C,D). H_2_O_2_ stimulation induced strong green fluorescence in HUVECs, while BORA-loaded micelles showed visibly lower fluorescence than the H_2_O_2_ model group, qualitatively approaching that observed in untreated cells. This finding suggests lower intracellular ROS-associated fluorescence under the tested conditions.

The effects of the micelles on inflammatory cytokine production were subsequently investigated by measuring TNF-α and IL-1β levels in H_2_O_2_-stimulated HUVECs using ELISA (Figure 7E,F). H_2_O_2_ stimulation markedly increased the secretion of both cytokines, whereas BORA-loaded micelles reduced TNF-α and IL-1β levels more effectively than free HBA, atRA, or their combination, with greater reductions observed at the higher micelle concentration. These results suggest that micelle treatment was associated with improved cell viability, lower intracellular ROS-associated fluorescence, and reduced TNF-α and IL-1β concentrations. Moreover, as oxidative stress can promote inflammatory activation in endothelial cells [39,40], the decreased TNF-α and IL-1β levels may be associated with attenuated oxidative stress following micelle treatment.

Overall, P2-Mal-PEG-b-PAsp/BORA micelle treatment was associated with improved HUVEC viability, lower ROS-associated fluorescence, and reduced TNF-α and IL-1β levels following H_2_O_2_ exposure.

### 3.5. In Vivo Evaluation in FeCl_3_-Induced Thrombosis

The preliminary in vivo effects of P2-Mal-PEG-b-PAsp/BORA micelles were evaluated in a FeCl_3_-induced carotid artery thrombosis model in SD rats. FeCl_3_ treatment produced darkening and gross morphological changes at the treated vascular segment, consistent with thrombotic vascular injury (Figure S4).

**Figure 8:**
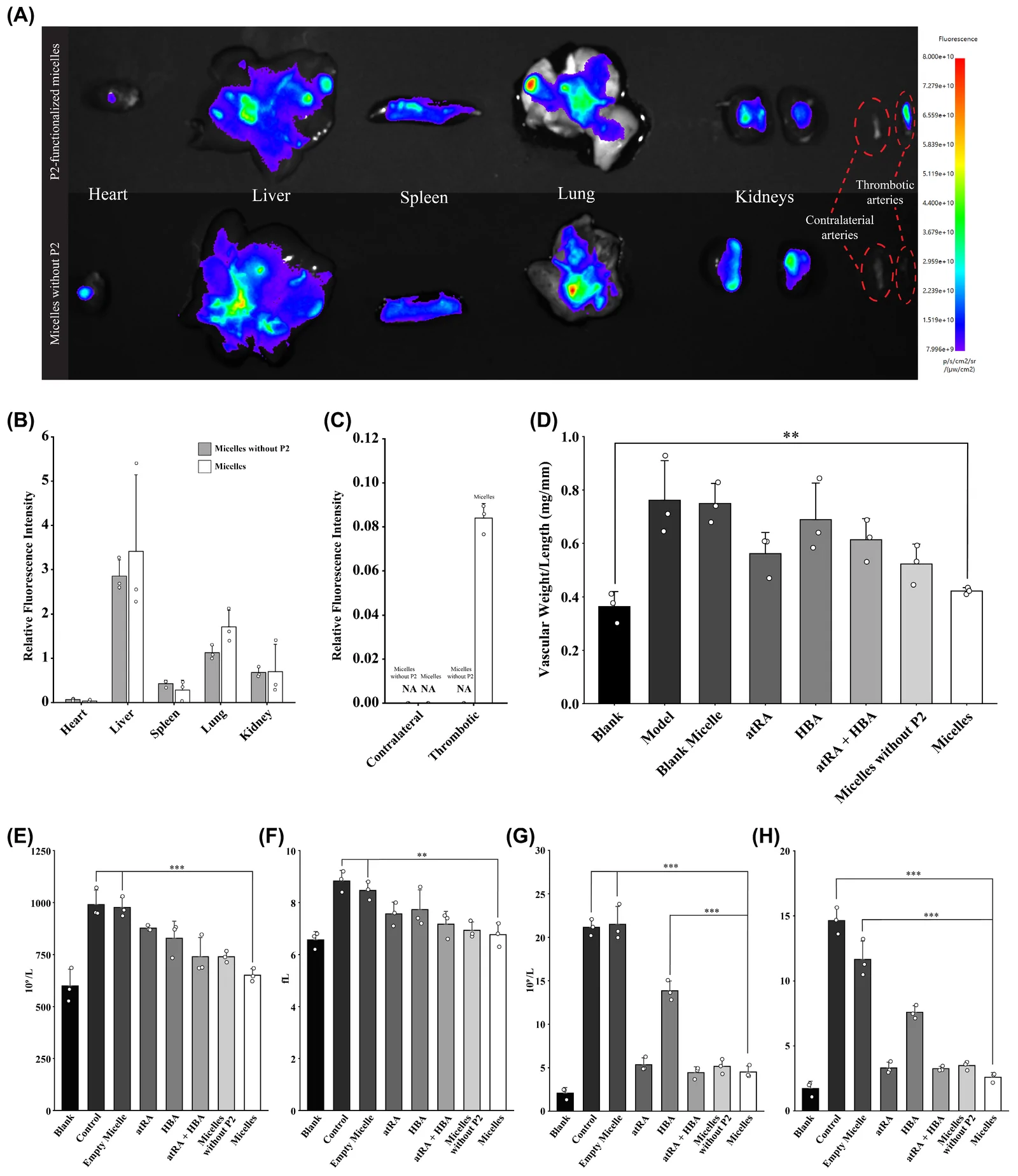
(**A**) Representative ex vivo fluorescence images of the heart, liver, spleen, lungs, kidneys, contralateral carotid artery, and thrombotic carotid artery collected from rats administered Cy5.5-labeled P2-functionalized micelles (upper row) or micelles without P2 (lower row). (**B**) Quantification of relative fluorescence intensity in the major organs. (**C**) Quantification of relative fluorescence intensity in the contralateral and thrombotic carotid arteries; NA indicates that the fluorescence signal was below the detection limit. (**D**) Weight-to-length ratios of the excised vascular segments. (**E**–**H**) Hematological indices after the indicated treatments, including platelet count (**E**), mean platelet volume (**F**), white blood cell count (**G**), and lymphocyte count (**H**). Data are presented as mean ± SD, with individual data points representing separate animals (n = 3 per group). Statistical significance was assessed using two-way ANOVA followed by Šídák’s multiple-comparisons test for the organ biodistribution data in (**B**), a two-tailed paired Student’s *t*-test for the comparison between contralateral and thrombotic arteries in (**C**), and one-way ANOVA followed by Tukey’s multiple-comparisons test for the multigroup data in (**D**–**H**). ** *p* < 0.01; *** *p* < 0.001. For the principal in vivo outcomes shown in (**D**–**H**), effect sizes were expressed as unstandardized mean differences (MDs; complete micelles minus the indicated comparator), together with Tukey-adjusted simultaneous 95% confidence intervals (CIs).

To assess the biodistribution and thrombus-associated localization of the micelles, Cy5.5-labeled micelles without P2 and fully P2-functionalized micelles were administered intravenously, followed by ex vivo fluorescence imaging of the major organs and carotid arteries (Figure 8A,B). Individual ex vivo fluorescence images from all rats receiving P2-functionalized or P2-free micelles are provided in Figures S5 and S6, respectively. Both formulations exhibited predominant fluorescence in the liver, consistent with the hepatic clearance typically observed for intravenously administered nanocarriers via the mononuclear phagocyte system [32,33]. Appreciable pulmonary fluorescence was also detected in both groups, whereas signals in the other organs were comparatively lower. Quantitatively, no statistically significant differences in organ-specific fluorescence were observed between the two formulations. The mean differences in relative fluorescence intensity, calculated as complete micelles minus micelles without P2, were −0.032 for the heart (Šidák-adjusted 95% CI, −1.450 to 1.385), 0.562 for the liver (95% CI, −0.856 to 1.979), −0.144 for the spleen (95% CI, −1.561 to 1.274), and 0.582 for the lungs (95% CI, −0.836 to 1.999); all confidence intervals included zero (Figure 8B). Given that similarly elevated pulmonary signals were observed for both formulations, this signal is unlikely to be specifically attributable to P2-mediated targeting. Instead, it may reflect transient retention in the pulmonary circulation, residual fluorescent material within the non-perfused lung vasculature, and the fact that Cy5.5 fluorescence does not necessarily correspond to intact micelles [41,42,43,44,45].

Following administration of the complete micelles, the thrombotic carotid artery showed a mean relative fluorescence intensity of 0.0840, whereas the corresponding signals in the contralateral arteries were below the applied fluorescence threshold and were recorded as zero. The paired mean difference between the thrombotic and contralateral arteries was 0.0840 (95% CI, 0.0675 to 0.1005; *p* = 0.0021) (Figure 8C). In rats receiving micelles without P2, the thrombotic-artery signals were also below the applied fluorescence threshold in all three animals. A thrombus-to-contralateral ratio was not calculated because the contralateral values were below the detection threshold. Together with the in vitro fibrin-binding and thrombus-association findings, these results support preferential association of the P2-functionalized formulation with the thrombotic vascular segment under the applied experimental and imaging conditions.

The treatment effects of the designed micelles were further evaluated using the weight-to-length ratio of the excised vascular segments. The complete P2-functionalized micelle group had a mean vascular weight-to-length ratio of 0.422 mg/mm, compared with 0.761 mg/mm in the model group (MD, −0.339 mg/mm; Tukey-adjusted 95% CI, −0.599 to −0.079) and 0.749 mg/mm in the blank-micelle group (MD, −0.327 mg/mm; 95% CI, −0.587 to −0.067). Although the mean ratio was also numerically lower than that in the micelles-without-P2 group (0.523 mg/mm), the corresponding confidence interval included zero (MD, −0.101 mg/mm; 95% CI, −0.361 to 0.160) (Figure 8D). Gross photographs of the harvested carotid artery specimens from all treatment groups are provided in Figure S7.

Hematological analysis showed that the complete-micelle group had a lower mean platelet count than the model group (650.7 versus 990.7 × 10^9^/L; MD, −340.0 × 10^9^/L; 95% CI, −515.3 to −164.7) and the blank-micelle group (650.7 versus 976.7 × 10^9^/L; MD, −326.0 × 10^9^/L; 95% CI, −501.3 to −150.7). The mean platelet count was also numerically lower than that in the micelles-without-P2 group (739.3 × 10^9^/L), although the corresponding confidence interval included zero (MD, −88.7 × 10^9^/L; 95% CI, −264.0 to 86.6) (Figure 8E). Mean platelet volume was lower in the complete-micelle group than in the model group (6.77 versus 8.83 fL; MD, −2.07 fL; 95% CI, −3.38 to −0.76) and the blank-micelle group (6.77 versus 8.47 fL; MD, −1.70 fL; 95% CI, −3.01 to −0.39). By contrast, the confidence interval for the comparison with micelles without P2 included zero (MD, −0.17 fL; 95% CI, −1.48 to 1.14) (Figure 8F).

The complete-micelle group additionally had a lower mean white blood cell count than the model group (4.51 versus 21.15 × 10^9^/L; MD, −16.64 × 10^9^/L; 95% CI, −19.66 to −13.63), the HBA group (4.51 versus 13.84 × 10^9^/L; MD, −9.34 × 10^9^/L; 95% CI, −12.35 to −6.32), and the blank-micelle group (4.51 versus 21.50 × 10^9^/L; MD, −16.99 × 10^9^/L; 95% CI, −20.01 to −13.97). The comparison with micelles without P2 was not statistically significant, as the confidence interval included zero (MD, −0.65 × 10^9^/L; 95% CI, −3.66 to 2.37) (Figure 8G). Similarly, the mean lymphocyte count was lower in the complete-micelle group than in the model group (2.58 versus 14.64 × 10^9^/L; MD, −12.06 × 10^9^/L; 95% CI, −14.08 to −10.04) and the blank-micelle group (2.58 versus 11.66 × 10^9^/L; MD, −9.07 × 10^9^/L; 95% CI, −11.09 to −7.06). Although the mean lymphocyte count was numerically lower than that in the micelles-without-P2 group, the corresponding confidence interval again included zero (MD, −0.90 × 10^9^/L; 95% CI, −2.92 to 1.12) (Figure 8H). These findings describe treatment-associated changes in circulating hematological indices but do not directly establish changes in platelet activation or vascular inflammation.

### 3.6. Comparison with Related Nanoplatforms and Future Perspectives

Recent thrombosis-oriented nanoplatforms have combined fibrin targeting with stimulus-responsive or multimodal therapy. CREKA-functionalized, RBC-membrane-coated dextran-tirofiban nanoparticles (~177 nm) achieved H_2_O_2_-triggered drug release and enhanced thrombus localization, whereas CREKA-decorated LMWH-derived nanoparticles containing Tempol and linoleic acid (~175 nm) integrated anticoagulant, antioxidant, and anti-inflammatory effects [46,47]. Fibrin-targeted NIR-II nanoparticles have additionally enabled image-guided photothermal/NO therapy [48]. In comparison, our similarly sized micelles (~196 nm) integrate the screened P2 peptide with an atRA/HBA boronate prodrug in a PEG-b-PAsp carrier, providing a complementary fibrin-targeted and H_2_O_2_-responsive strategy for thrombosis treatment.

Building on the present findings, the exploratory in vivo evaluation was conducted in female rats (n = 3 per group); future studies involving larger, sex-balanced cohorts, guided by prospective power calculations and incorporating randomized, blinded assessments, would support more precise effect estimates and more informative evaluation of distributional and variance assumptions. Complementary cellular and flow-based models incorporating fibrin, platelets, thrombin, leukocytes, and physiological shear forces could further extend the H_2_O_2_-treated HUVEC findings and more closely represent the cellular and hemodynamic complexity of thrombosis. Further characterization of platelet activation, vascular inflammation, competitive binding, pharmacokinetics, circulation time, and systemic safety, together with batch-to-batch assessment and HPLC or LC-MS confirmation of BORA degradation products, would consolidate the mechanistic and translational foundation of this platform.

## 4. Conclusions

In this study, a P2-functionalized, H_2_O_2_-responsive retinoic acid prodrug micellar system was developed as a candidate formulation for attenuating thrombosis-associated oxidative and inflammatory responses. The candidate fibrin-binding peptide P2 (VTFIKC) was identified through computer-aided peptide screening, and its fibrin-binding and in vitro thrombus-association properties were supported by in vitro assays. Based on its binding affinity, low hemolytic activity, cytocompatibility, and stability under the tested conditions, P2 was selected as the ligand for micelle construction.

The H_2_O_2_-responsive prodrug BORA was synthesized and co-assembled with P2-Mal-PEG-b-PAsp to form P2-functionalized polymeric micelles. The resulting P2-Mal-PEG-b-PAsp/BORA micelles showed suitable nanoscale size, negative surface charge, defined morphology, and substantial apparent prodrug-loading content. Upon H_2_O_2_ exposure, the micelles showed changes in particle size distribution, accelerated retinoic acid release, and concentration-dependent H_2_O_2_ consumption, supporting their H_2_O_2_-responsive formulation design.

In vitro, micelle treatment was associated with improved HUVEC viability following H_2_O_2_ exposure, lower intracellular ROS-associated fluorescence, and reduced TNF-α and IL-1β concentrations. In a FeCl_3_-induced carotid artery thrombosis model, systemic administration of the micelles was associated with changes in platelet- and leukocyte-related hematological indices, and the micelles showed preferential accumulation in the thrombotic carotid artery.

Overall, in line with advances in targeted and stimuli-responsive nanodrug delivery [49,50], this study combined a newly screened candidate fibrin-binding peptide, an H_2_O_2_-responsive atRA prodrug, and a polymeric micellar carrier into a single formulation. Nowadays, mechanism-oriented vascular intervention and formulation-enabled cardiovascular drug delivery are increasingly being explored as complementary therapeutic strategies [51,52]. Unlike conventional antithrombotic strategies that mainly interfere with coagulation, platelet aggregation, or fibrinolysis systemically [5,53,54], this micelle modulated thrombosis-associated oxidative and inflammatory responses through P2-mediated fibrin-binding potential, H_2_O_2_-dependent release behavior, and H_2_O_2_ consumption. The current findings provide formulation and biological proof-of-concept for further investigation of this platform, while the small animal cohort, limited mechanistic endpoints, and incomplete in vivo distribution, pharmacokinetic, and safety characterization preclude definitive conclusions regarding thrombus-specific delivery or therapeutic efficacy.

## Data Availability

The raw data supporting the conclusions of this article will be made available by the corresponding authors upon reasonable request.

## Supplementary Materials

The following supporting information can be downloaded at: https://www.mdpi.com/article/10.3390/biomedicines14092015/s1.
