# Supplementary material for: Fibrin-Binding Peptide-Functionalized H2O2-Responsive Retinoic Acid Micelles for Attenuating Thrombosis-Associated Oxidative and Inflammatory Responses
Source: Biomedicines. 2026 Sep 8;14(9):2015. doi: 10.3390/biomedicines14092015 (PMC13604930; doi:10.3390/biomedicines14092015)
Supplement: Supplementary file 1 [file biomedicines-14-02015-s001.zip › biomedicines-4509111-supplementary.pdf]

Fibrin-Binding Peptide-Functionalized H_2O_2-Responsive Retinoic Acid Micelles for Attenuating Thrombosis-Associated Oxidative and Inflammatory Responses

Junkai Zhao 1, Mengting Xie 1, Jianghao Yu 1, Ran Yan 1, * and Yue Wang 1, *

1 Key Laboratory of Biomedical Functional Materials, School of Science, China Pharmaceutical University, No. 639 Longmian Avenue, Jiangning District, Nanjing 211198, China; 2020220547@stu.cpu.edu.cn (J.Z.)

*These authors are corresponding authors

* Correspondence: frankyan@cpu.edu.cn (R.Y.); zwy_1115@cpu.edu.cn (Y.W.)

Supporting Information

Candidate Peptides	Docking score	Asp320	Asp291	Asn308	Lys302	Trend
P1-MRFIEC	-9.803	√	√	-	√	Good
P2-VTFIKC	-9.597	√	-	√	-	Good
P3-MRFVKS	-9.73	√	√	√	-	Good
P4-MGFIKG	-9.631	√	√	-	√	Good
P5-MTLVKS	-9.771	-	-	√	-	Good

Table S1. The scores of candidate peptides and their interactions with key amino acids

Block Ratio	Feed Ratio	Particle Size	PDI	Zeta Potential
Mal-PEG45-b-PAsp10	1:40	121.5	0.272	-5.11
Mal-PEG45-b-PAsp15	1:80	153	0.388	-9.59
Mal-PEG45-b-PAsp20	1:160	174.6	0.183	-21.1

Table S2. Particle size, polydispersity index (PDI), and zeta potential of Mal-PEG-b-Pasp with different block ratios and feed ratios. The feed ratio refers to the molar feed ratio of Mal-PEG-NH_2 to BLA-NCA used during polymer synthesis.

Synthesis of Components for Micelle Construction

Synthesis of BORA Prodrug

4-(Bromomethyl)phenylboronic acid (1.00 g, 4.65 mmol) and trimethylolethane (1.10 g, 6.70 mmol) were dissolved in THF (5 mL) and stirred at room temperature for 4 h. After solvent removal under reduced pressure, DCM (10 mL) was added to the crude white product, and the mixture was filtered through a 200 nm membrane to remove excess trimethylolethane. This purification step was repeated three times, followed by solvent evaporation to obtain the boronate linker.

To synthesize BORA, all-trans retinoic acid (atRA, 2.00 g, 6.66 mmol) and cesium carbonate (2.00 g) were dissolved in DMF (10.0 mL) and stirred at room temperature for 15 min to activate the carboxyl group of atRA. Boronate linker (2.00 g, 6.71 mmol) in DMF (5.00 mL) was then added, and the reaction was stirred at 40 °C for 24 h. The mixture was extracted with ethyl acetate and water while maintaining the pH at approximately 7.5. The organic phase was dried over anhydrous sodium sulfate, concentrated under reduced pressure, and purified by column chromatography using dichloroethane/methanol (10/1, v/v) as the eluent to yield BORA as a pale-yellow powder.

Synthesis of Mal-PEG-b-PAsp

β-Benzyl-L-aspartate N-carboxyanhydride (BLA-NCA) was first synthesized as the monomer for ring-opening polymerization. Benzyl-L-aspartate (4.00 g, 17.9 mmol) was dispersed in anhydrous THF (20 mL) and stirred at 45 °C for approximately 15 min. Triphosgene (2.00 g, 6.72 mmol) in anhydrous THF (10 mL) was then added dropwise through a constant-pressure dropping funnel, and the reaction was stirred at 45 °C for 2 h until the milky suspension became clear. The crude product was precipitated with approximately 150 mL n-hexane, collected by vacuum filtration, redissolved in THF, recrystallized with n-hexane three times, and dried under vacuum at 40 °C to obtain BLA-NCA as a white solid.

Mal-PEG-b-PAsp was synthesized by NCA ring-opening polymerization using Mal-PEG-NH_2 as the macroinitiator. Mal-PEG-NH_2 (200 mg, 0.02 mmol) was dissolved in anhydrous DCM/DMF (12 mL, 3:1, v/v), transferred to a Schlenk-type tube, and mixed with dried BLA-NCA (2.0 g). The reaction was conducted under nitrogen at 40 °C for 72 h under anhydrous and oxygen-free conditions. The product was precipitated with excess anhydrous diethyl ether, transferred into 10 mL centrifuge tubes, centrifuged at 800 g for 5 min, and dried under vacuum for 6 h to remove residual diethyl ether. The obtained solid was dissolved in DMF and dialyzed against deionized water using a dialysis bag with a molecular weight cut-off of 3500 Da for three days, with water changed every 4 h. After dialysis, the solution was pre-frozen at -80 °C and lyophilized for 24 h to obtain purified PEG-b-PBLA.

To remove the benzyl protecting groups, PEG-b-PBLA was dissolved in dichloroacetic acid (10 mL), followed by addition of an equal volume of 33% hydrobromic acid/acetic acid solution (10 mL). The mixture was stirred at room temperature for 6 h, and the deprotected polymer was precipitated with anhydrous diethyl ether. The precipitate was collected by centrifugation at 800 g for 5 min, dried under vacuum for 6 h, dissolved in a small amount of

DMF, and dialyzed against deionized water using a 3500 Da dialysis bag for three days, with water changed every 4 h. The solution was then pre-frozen at −80 °C and lyophilized to afford purified Mal-PEG-b-PAsp as a white solid.

Different polymerization degrees of Mal-PEG-b-PAsp were obtained by adjusting the feed molar ratio of Mal-PEG-NH_2 to BLA-NCA. Feed ratios of 1:40, 1:80, and 1:160 were investigated to prepare polymer backbones with different block ratios and molecular weights. The block ratio of Mal-PEG-b-PAsp was calculated from the grafting ratio of polyaspartic acid based on the 2.80 ppm signal.

Synthesis of P2-Mal-PEG-b-PAsp

P2-Mal-PEG-b-PAsp was synthesized through a thiol-maleimide Michael addition reaction between the terminal cysteine residue of P2 and the maleimide group of Mal-PEG-b-PAsp. Mal-PEG-b-PAsp (20 mg) was dissolved in anhydrous DMF (2 mL). Separately, the candidate peptide P2 (10 mg) was dissolved in DMF (5 mL) under gentle stirring. The Mal-PEG-b-PAsp solution was then slowly added dropwise into the peptide solution, and the reaction mixture was stirred at room temperature for 24 h. After completion of the reaction, the mixture was transferred into a dialysis bag with a molecular weight cut-off of 3.5 kDa and dialyzed against deionized water to remove excess peptide and DMF. The dialyzed solution was pre-frozen at −80 °C and subsequently lyophilized to obtain P2-Mal-PEG-b-PAsp.

Characterizations of Components for Micelle Construction

Characterization of boronate linker (**Figure S7**): ^{1}H NMR (400 MHz, DMSO-d6) δ 7.66 (d, j = 8.0 Hz, 2H), 7.40 (d, j = 8.1 Hz, 2H), 4.80 (d, j = 36.3 Hz, 1H), 4.69 (s, 2H), 3.94 (d, j = 10.8 Hz, 2H), 3.75 (d, j = 10.8 Hz, 2H), 3.36 (s, 2H), 0.87 (s, 3H).

Characterization of BORA (**Figure S1 & S2**): ^{1}H NMR (300 MHz, DMSO-d6) δ 7.69 (d, j = 7.9 Hz, 2H), 7.35 (d, j = 8.0 Hz, 2H), 5.92 (s, 1H), 5.14 (s, 2H), 3.93 (s, 2H), 3.78 (s, 2H). ESI-MS m/z： 557.28343 $[M+Na]^+$.

Characterization of BLA-NCA (**Figure S8**): ^{1}H NMR (300 MHz, DMSO-d6) δ 9.01 (s, 1H), 7.39 (s, 5H), 5.14 (s, 2H), 4.70 (t, j = 5.0 Hz, 1H), 3.09 (dd, j = 17.8, 4.8 Hz, 1H), 2.90 (dd, j = 17.8, 4.2 Hz, 1H). The signal at 9.01 ppm was assigned to the amide NH proton in the cyclic imide structure, indicating the successful cyclization of BLA-NCA.

Characterization of Mal-PEG-b-PBLA and Mal-PEG-b-PAsp (**Figures S9-S12**): As shown in **Figure S9**, the chemical shift at 3.51 ppm corresponds to the characteristic peak of the polyethylene glycol segment, and the aromatic protons on the benzyl protecting groups appear at 7.30 ppm. At the same time, the characteristic peak of the maleimide group can be observed at 8.20 ppm. These signals indicate that BLA-NCA was successfully polymerized onto Mal-PEG-NH_2. ^{1}H NMR spectra of Mal-PEG45-b-PAsp10, Mal-PEG45-b-PAsp15, and Mal-PEG45-b-PAsp20 are shown in **Figures S10-12**. The disappearance of the aromatic peak at 7.30 ppm indicates successful removal of the benzyl protecting groups. The characteristic peak of PEG remains at 3.51 ppm, while the characteristic peaks of polyaspartic acid are observed at 2.80 ppm (-CH-CH_2-COOH) and 5.1-5.2 ppm (-NHCHCO-). By integrating the PEG methylene signal at 3.51 ppm relative to the PAsp methine signal at 5.1-5.2 ppm, the degrees of polymerization of the PAsp blocks were calculated to be approximately 10, 15, and 20, respectively. These results confirmed the successful preparation of Mal-PEG_{45}-b-$PAsp_{10}$, Mal-PEG_{45}-b-$PAsp_{15}$, and Mal-PEG_{45}-b-$PAsp_{20}$.

Characterization of P2-Mal-PEG-b-Pasp (**Figure S3**): The disappearance of the maleimide proton signal at 8.20 ppm in the ^{1}H NMR spectrum confirmed the successful conjugation of the P2 peptide to Mal-PEG-b-PAsp.

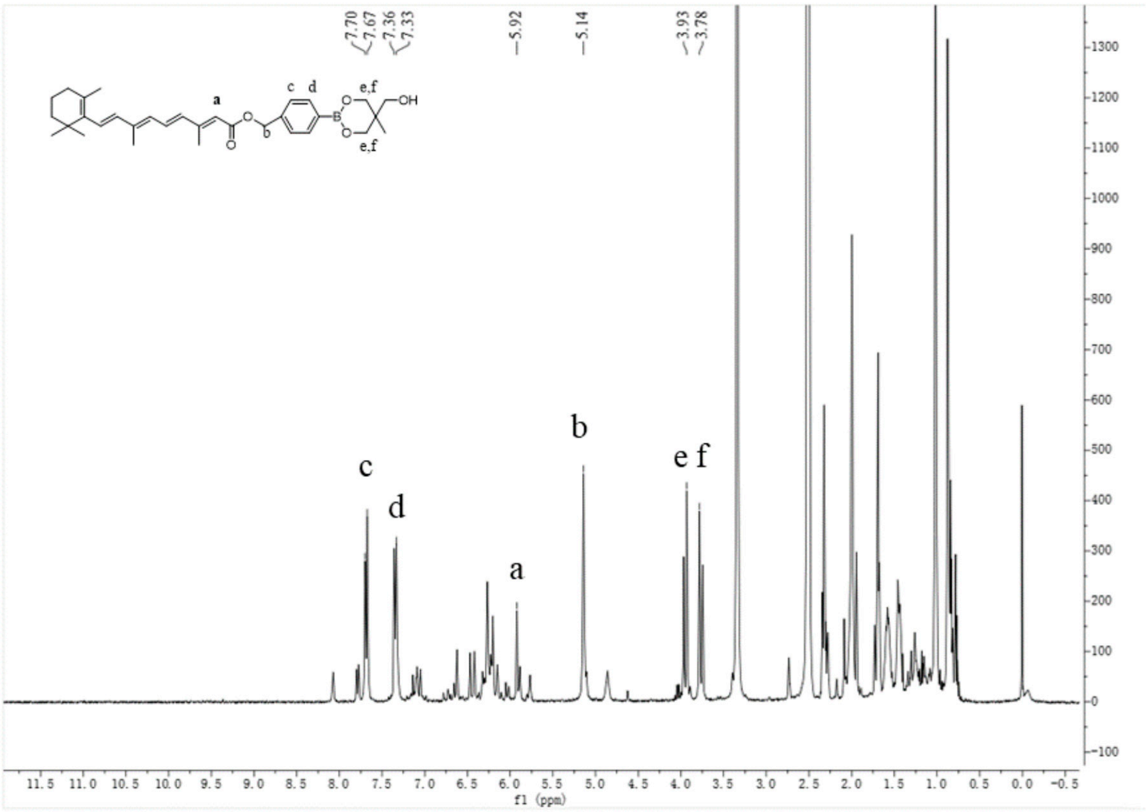

Figure S1. ^{1}H-NMR Spectrum of BORA

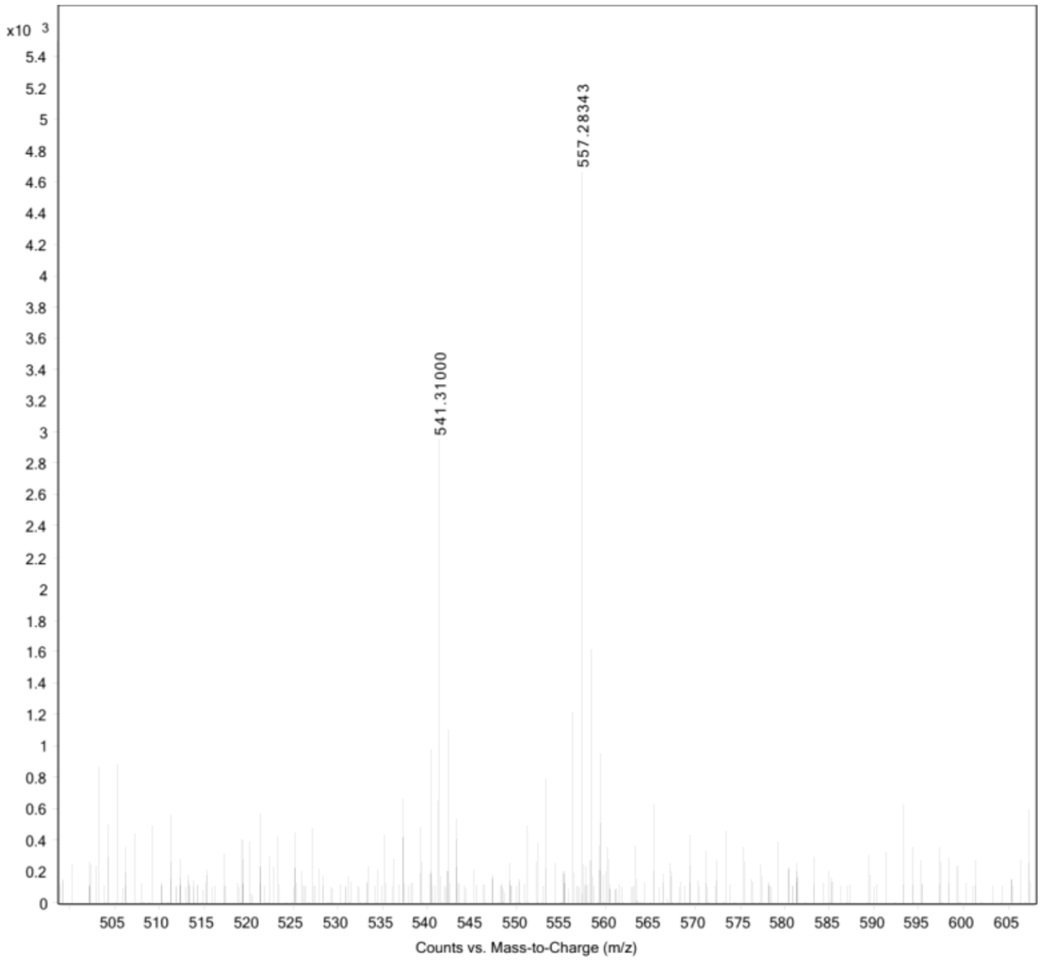

Figure S2. MS Spectrum of BORA

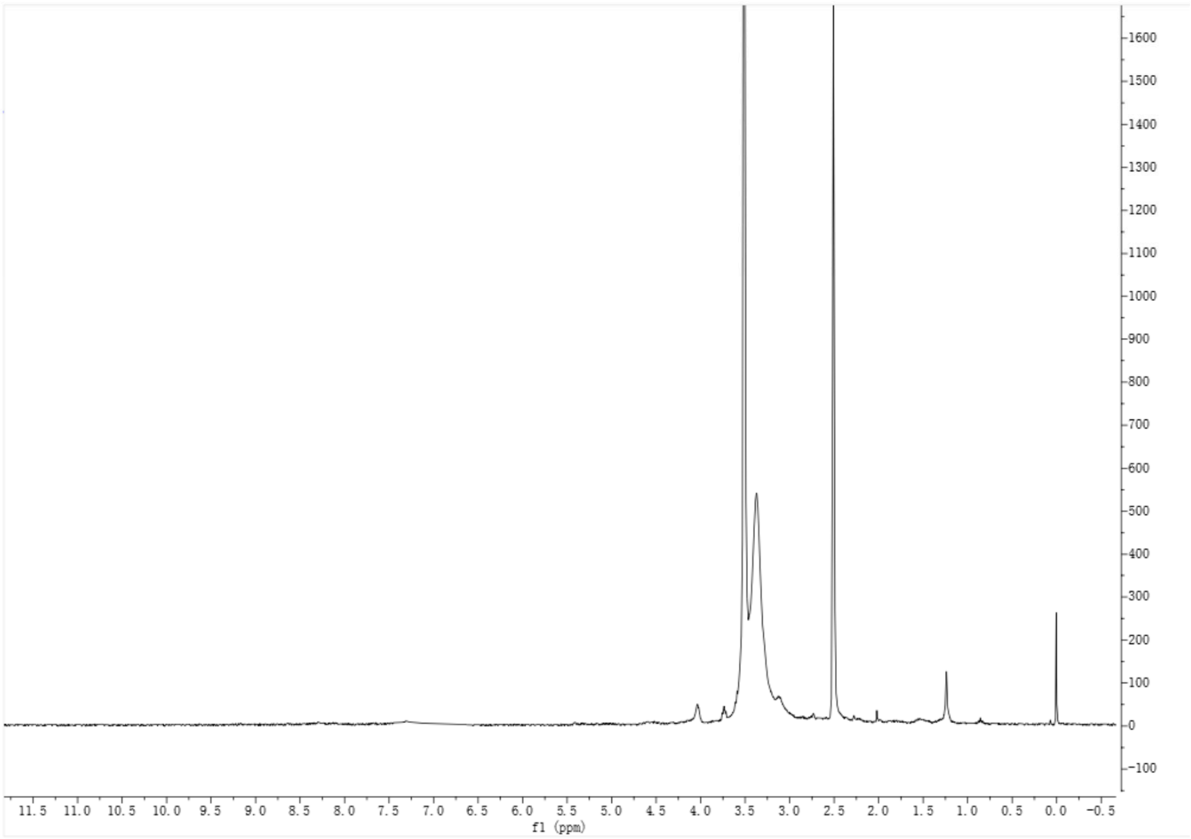

Figure S3. ^{1}H-NMR Spectrum of P2-Mal-PEG-b-PAsp

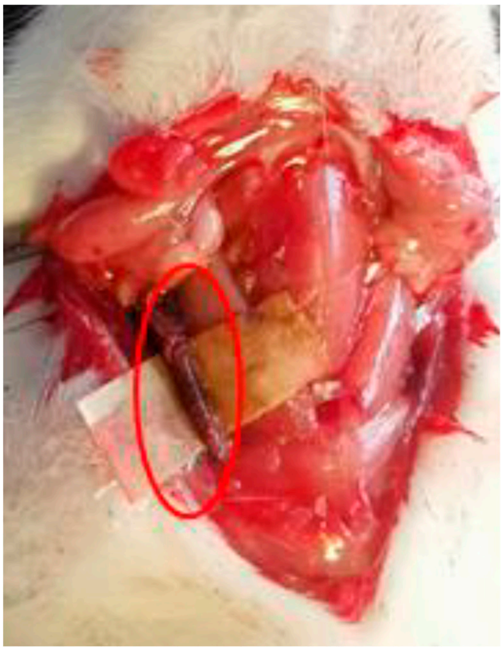

Figure S4. Representative gross photograph of the $FeCl_3$-induced rat carotid artery thrombosis model, with the $FeCl_3$-treated thrombotic segment highlighted.

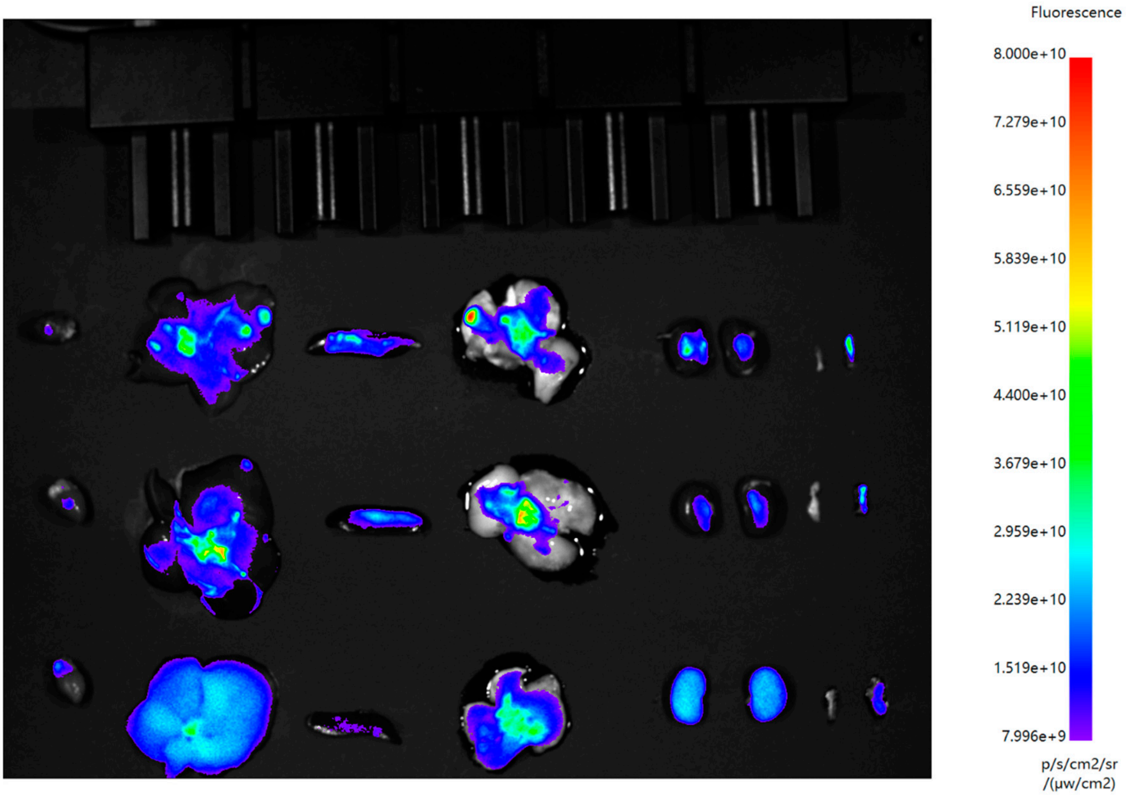

Figure S5. Individual ex vivo fluorescence images of major organs and carotid arteries from rats administered Cy5.5-labeled P2-functionalized BORA-loaded micelles. From left to right: heart, liver, spleen, lung, bilateral kidneys, contralateral carotid artery, and $FeCl_3$-induced thrombotic carotid artery. Each row represents one rat ($n = 3$). The color scale represents radiant efficiency ([p/s/cm²/sr]/[μW/cm²]).

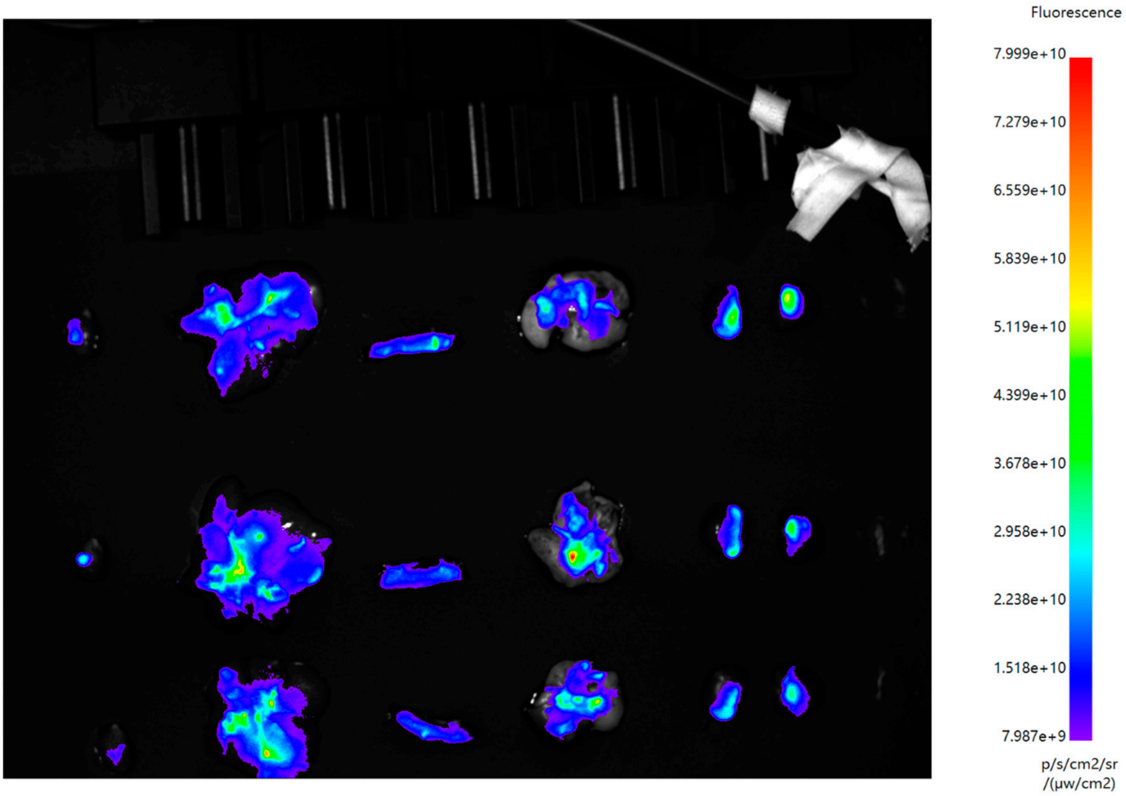

Figure S6. Individual ex vivo fluorescence images of major organs and carotid arteries from rats administered Cy5.5-labeled P2-free BORA-loaded micelles. From left to right: heart, liver, spleen, lung, bilateral kidneys, contralateral carotid artery, and $FeCl_3$-induced thrombotic carotid artery. Each row represents one rat (n = 3). The color scale represents radiant efficiency ([p/s/cm²/sr]/[μW/cm²]).

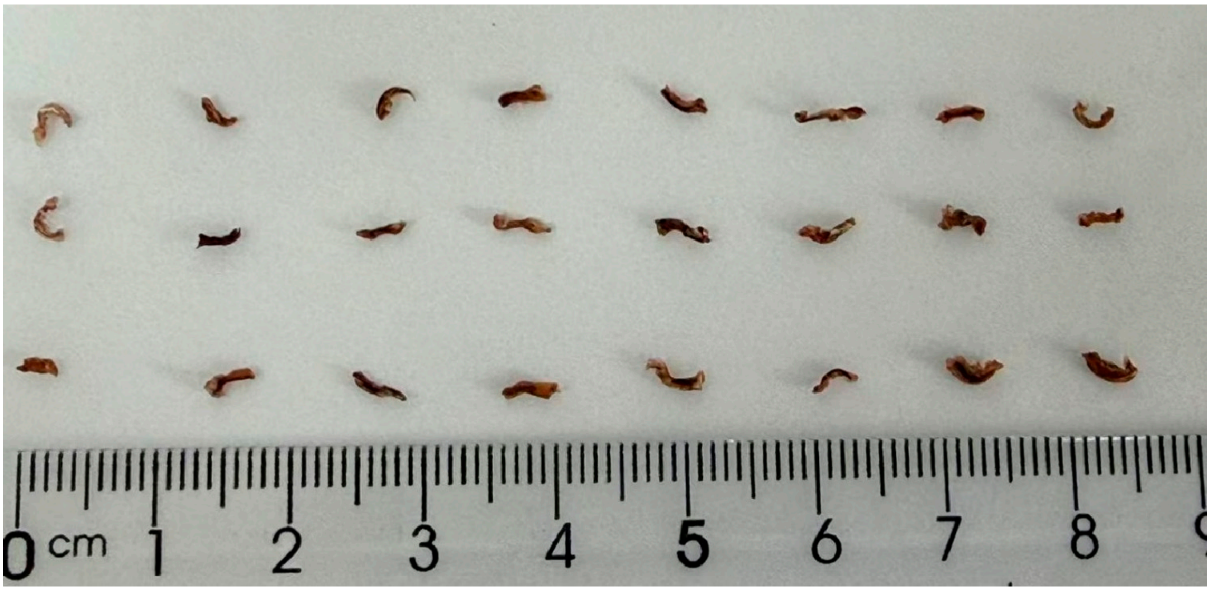

Figure S7. Gross appearance of harvested carotid artery specimens from the blank group and rats with $FeCl_3$-induced carotid artery thrombosis following different treatments. From left to right: blank, control, drug-free micelle, all-trans retinoic acid (atRA), p-hydroxybenzyl alcohol (HBA), atRA + HBA, P2-free micelle, and P2-functionalized micelle groups. Three specimens from individual rats are shown per group (n = 3). Ruler graduations are in centimeters.

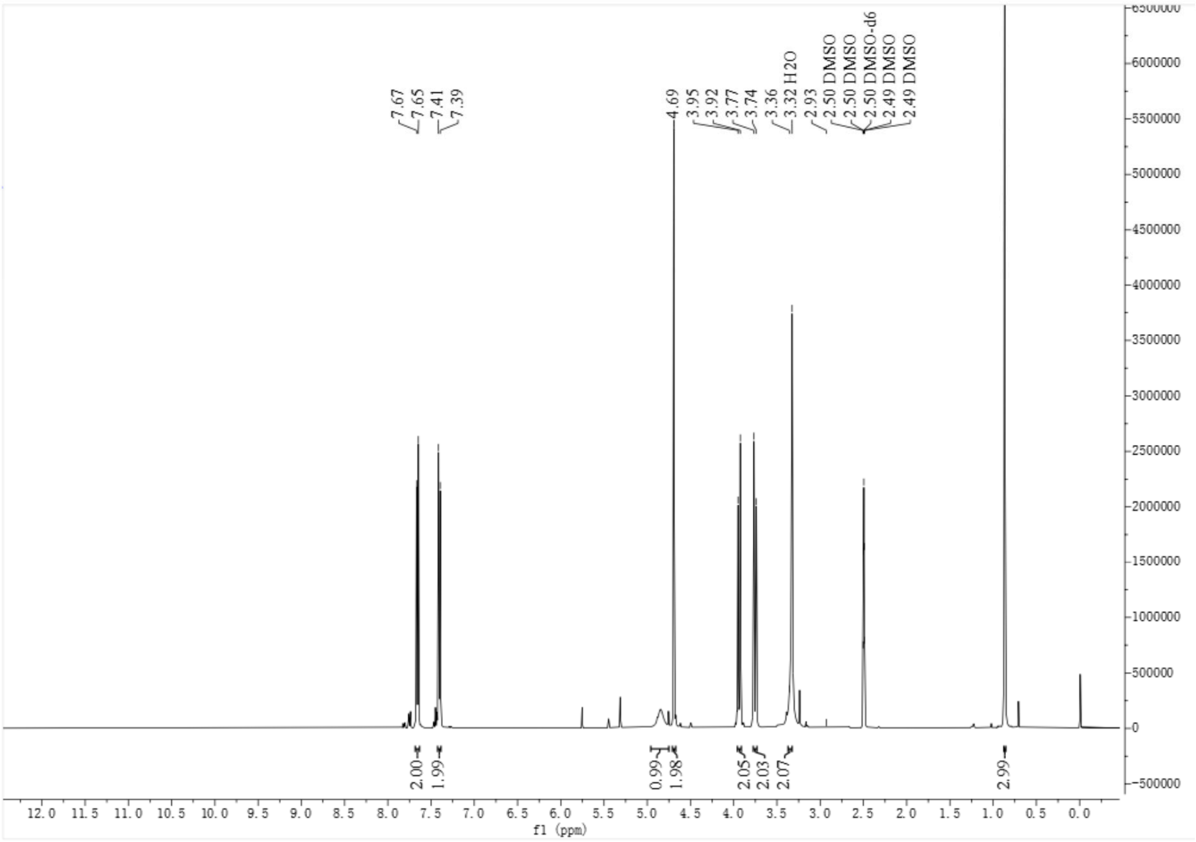

Figure S8. ¹H-NMR Spectrum of Boronate linker

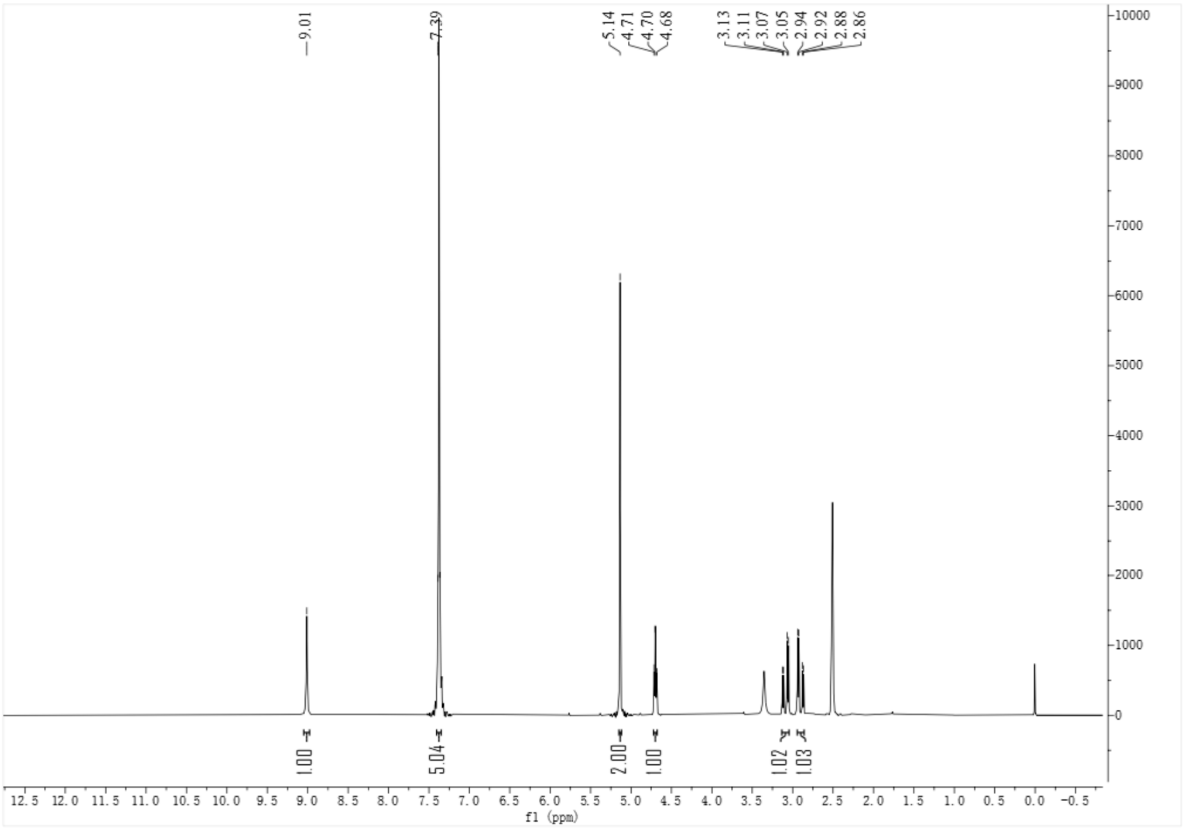

Figure S9. ^{1}H-NMR Spectrum of BLA-NCA

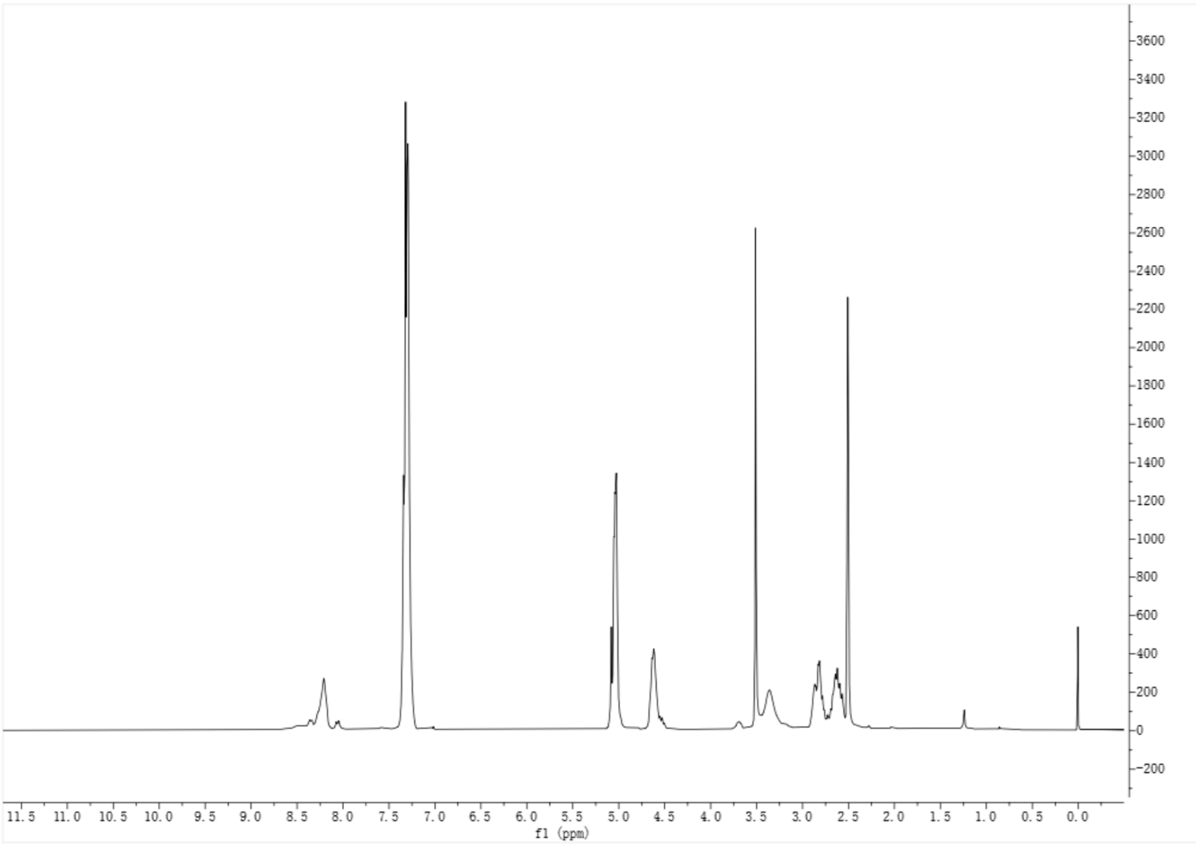

Figure S10. ^{1}H-NMR Spectrum of Mal-PEG-b-PBLA

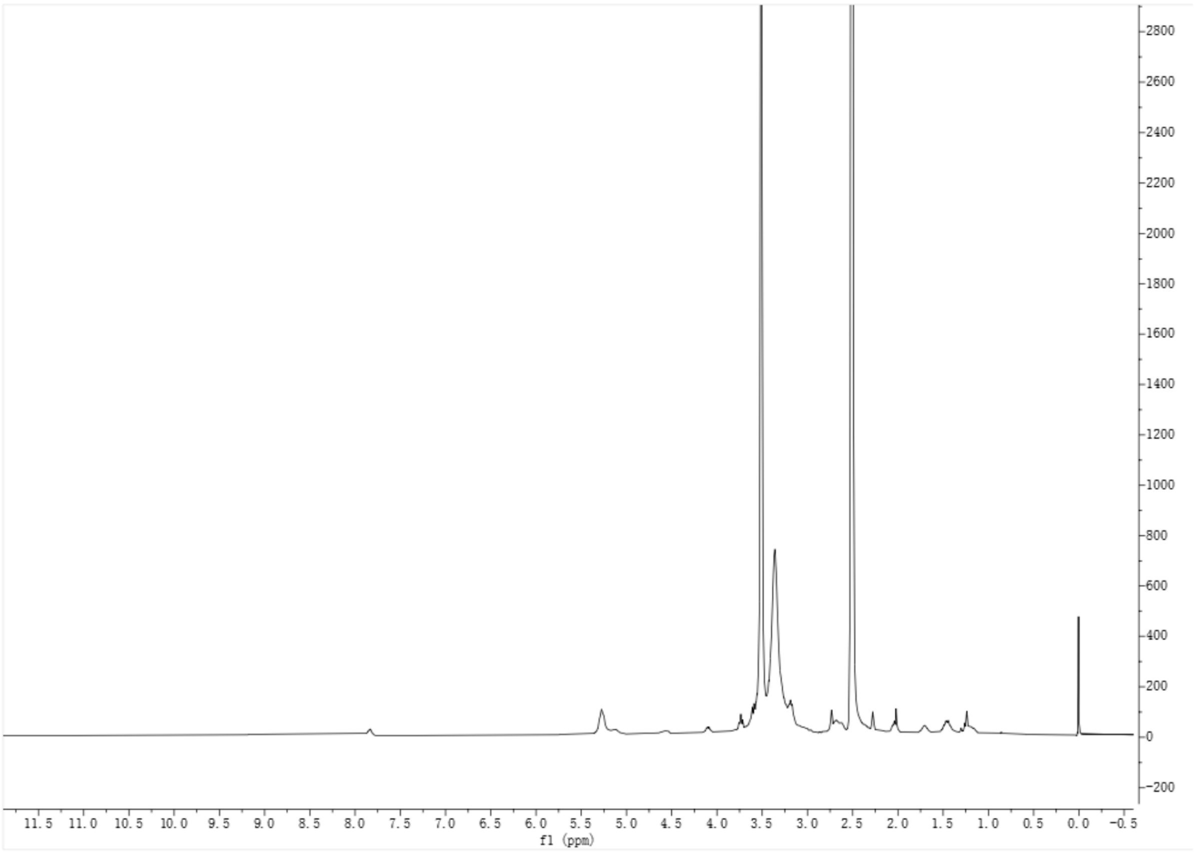

Figure S11. ^{1}H-NMR Spectrum of Mal-PEG45-b-PAsp10

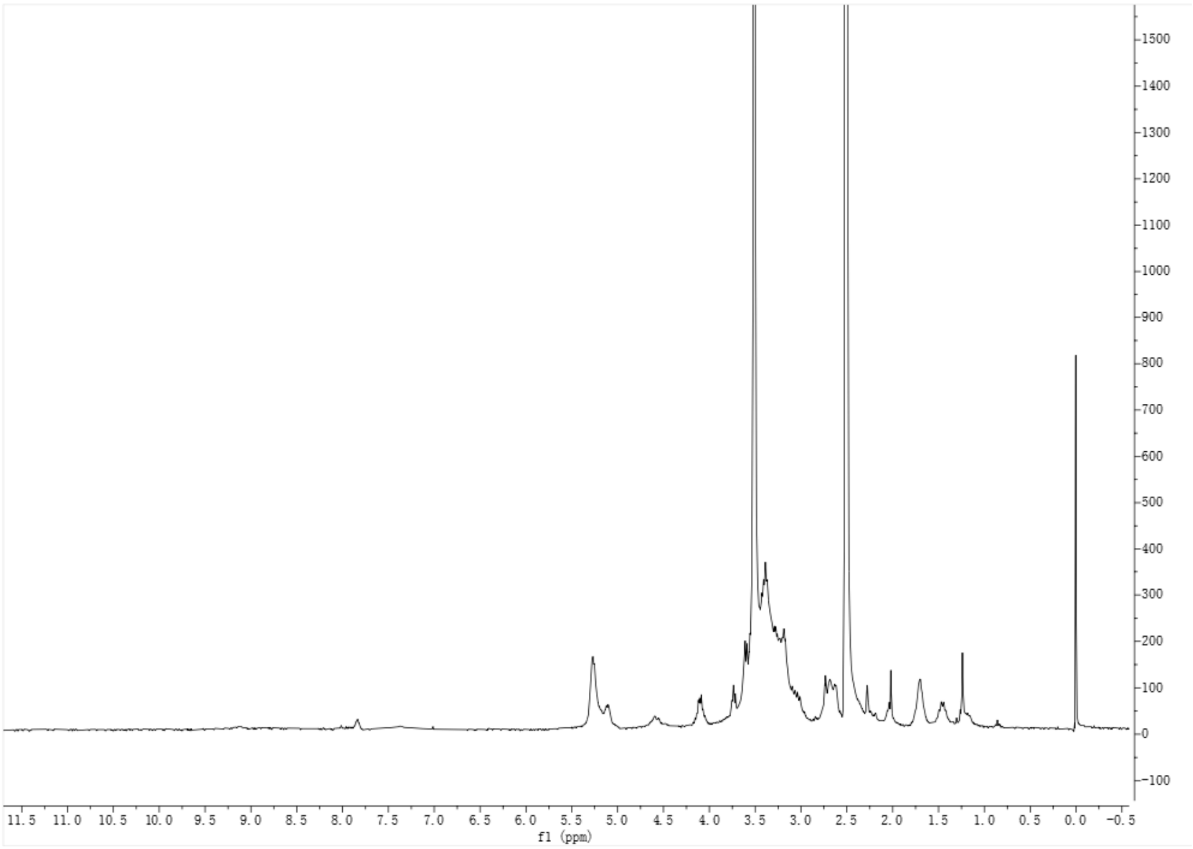

Figure S12. ^{1}H-NMR Spectrum of Mal-PEG45-b-PAsp15

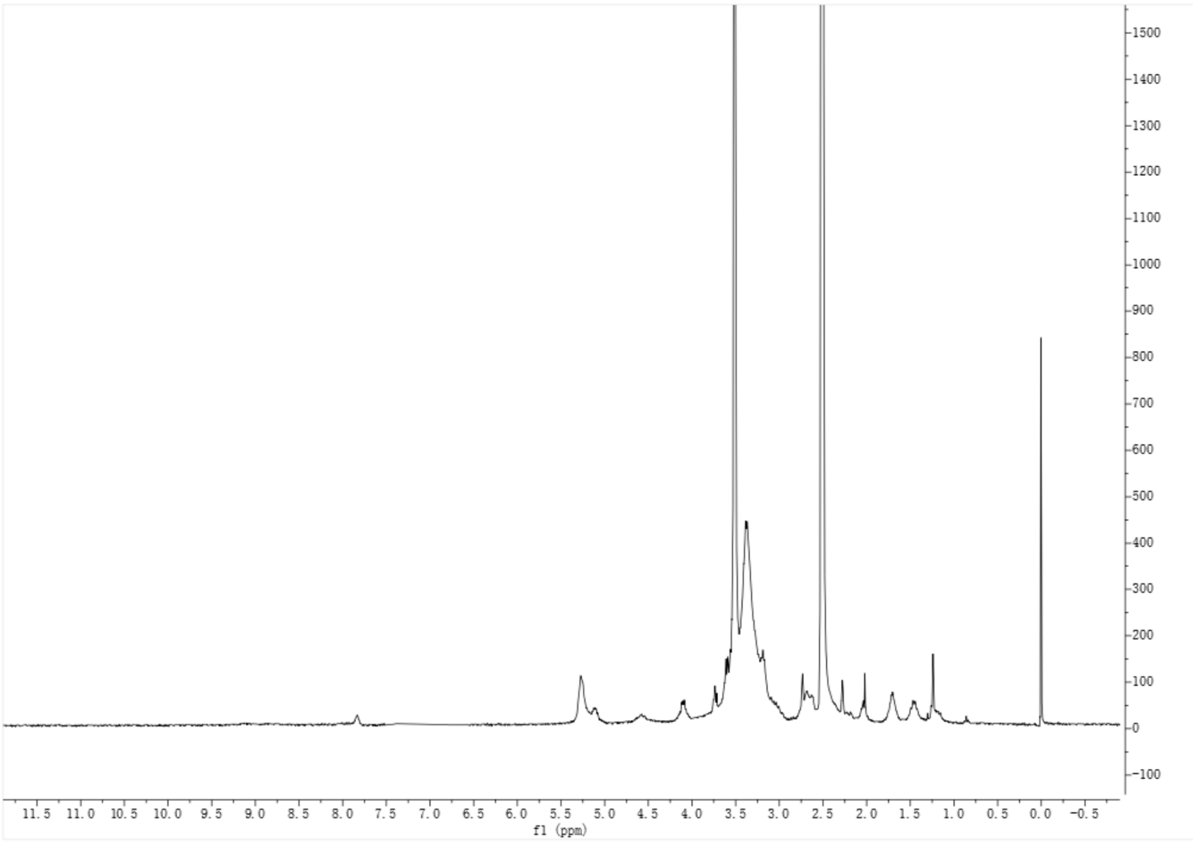

Figure S13. ^{1}H-NMR Spectrum of Mal-PEG45-b-PAsp20
